# Ordered clustering of single atomic Te vacancies in atomically thin PtTe_2_ promotes hydrogen evolution catalysis

**DOI:** 10.1038/s41467-021-22681-4

**Published:** 2021-04-21

**Authors:** Xinzhe Li, Yiyun Fang, Jun Wang, Hanyan Fang, Shibo Xi, Xiaoxu Zhao, Danyun Xu, Haomin Xu, Wei Yu, Xiao Hai, Cheng Chen, Chuanhao Yao, Hua Bing Tao, Alexander G. R. Howe, Stephen J. Pennycook, Bin Liu, Jiong Lu, Chenliang Su

**Affiliations:** 1 SZU-NUS Collaborative Center and International Collaborative Laboratory of 2D Materials for Optoelectronic Science and Technology of Ministry of Education, Institute of Microscale Optoelectronics, Shenzhen University, Shenzhen, Guangdong, China; 2 Department of Chemistry, National University of Singapore, Singapore, Singapore; 3 Frontiers Science Center for Flexible Electronics, Xi’an Institute of Flexible Electronics (IFE) and Xi’an Institute of Biomedical Materials & Engineering, Northwestern Polytechnical University, Xi’an, China; 4 School of Electrical Engineering and Automation, Wuhan University, Wuhan, China; 5 Institute of Chemical and Engineering Sciences, Agency for Science, Technology and Research (A*STAR), Singapore, Singapore; 6 Department of Materials Science and Engineering, National University of Singapore, Singapore, Singapore; 7 School of Chemical and Biomedical Engineering, Nanyang Technological University, Singapore, Singapore

**Keywords:** Electrocatalysis, Materials for energy and catalysis, Electrocatalysis

## Abstract

Exposing and stabilizing undercoordinated platinum (Pt) sites and therefore optimizing their adsorption to reactive intermediates offers a desirable strategy to develop highly efficient Pt-based electrocatalysts. However, preparation of atomically controllable Pt-based model catalysts to understand the correlation between electronic structure, adsorption energy, and catalytic properties of atomic Pt sites is still challenging. Herein we report the atomically thin two-dimensional PtTe_2_ nanosheets with well-dispersed single atomic Te vacancies (Te-SAVs) and atomically well-defined undercoordinated Pt sites as a model electrocatalyst. A controlled thermal treatment drives the migration of the Te-SAVs to form thermodynamically stabilized, ordered Te-SAV clusters, which decreases both the density of states of undercoordinated Pt sites around the Fermi level and the interacting orbital volume of Pt sites. As a result, the binding strength of atomically defined Pt active sites to H intermediates is effectively reduced, which renders PtTe_2_ nanosheets highly active and stable in hydrogen evolution reaction.

## Abstract (web summary)

Precisely regulating Pt catalytic sites is important and challenging. Herein the authors engineer the clustering of single atomic Te vacancies in atomically thin PtTe_2_ to optimize the electronic structure, adsorption energy, and catalytic performance of atomically defined Pt sites.

## Introduction

Global challenges, such as the increased energy demand, environmental pollution, and limited earth resources, are currently threatening sustained human development. Exploiting advanced materials and green technology, e.g., electrocatalysis, to convert sustainable resources (e.g., H_2_O, CO_2_, N_2_, and solar energy) into high value-added products (e.g., H_2_, O_2_, hydrocarbons, and NH_3_) is promising to address these problems^1^. For example, transition metal phosphides exhibit platinum (Pt)-like activity for hydrogen production via electrocatalytic water splitting^2–4^. However, up to now, rational design and preparation of advanced electrocatalysts with high activity and stability is still urgently required for practical applications, especially for Pt-based catalysts, which play versatile roles in energy-related electrocatalysis, such as alcohol oxidation reaction, hydrogen evolution/oxidation reaction (HER/HOR), and oxygen reduction reaction (ORR)^5^. To obtain excellent performance in these reactions, the catalytic behavior of atomic Pt sites, along with their local-structure environments needs to be deciphered. In this regard, over the past decades, a large variety of strategies have been developed to identify the decisive factors that influence the catalytic behaviors of Pt sites during various electrochemical reactions^6–16^. The corresponding results unveil that exposing undercoordinated Pt sites as well as optimizing the adsorption of the reaction intermediates (e.g., H^*^, O^*^, OH^*^, OOH^*^) to these sites is a desirable strategy to drastically enhance electrocatalytic activity^6–13^. For instance, acid-etched Pt-Ni alloys provide sufficient and accessible Pt sites, and the electronic and strain effects help to weaken the binding strength of Pt sites to oxygenated species, thus showing excellent ORR activities^6^. Although great progress has been achieved, simultaneously realizing the exposure and stabilization of undercoordinated Pt sites, as well as optimizing the relationship between electronic structure, adsorption energy, and catalytic properties of Pt sites at the atomic scale still remains a grand challenge, in part due to difficulties in precisely tailoring such kind of well-defined atomic Pt sites.

In this work, we design and prepare atomically thin two-dimensional (2D) PtTe_2_ nanosheets (NSs) with well-dispersed single atomic Te vacancies (Te-SAVs) by electrochemically exfoliating bulk PtTe_2_ crystals, in which large numbers of atomically defined undercoordinated and stabilized Pt sites are exposed. Consequently, the obtained 2D PtTe_2_ NSs have a well-defined structure and can serve as a Pt-based model catalyst, which are different from traditional Te or Pt-based noble metal materials. The following heat treatment causes migration of the random Te-SAVs to form ordered Te-SAV clusters. Both electrochemical measurements and density functional theory (DFT) calculations show that the heat treatment-induced migration of Te-SAVs in PtTe_2_ can effectively tailor the hydrogen adsorption energy (Δ*G*_H*_) on the undercoordinated Pt sites. Consequently, the PtTe_2_ NSs with ordered clusters of Te-SAVs exhibit much-enhanced HER activity with an exceptionally low onset potential (~0 mV), overpotential (*η*, 22 mV at 10 mA cm^−2^) and Tafel slope (29.9 mV per dec^−1^). Furthermore, after 20,000 continuous potential cycles and chronopotentiometry test at high current densities (200 mA cm^−2^) for 24 h, the catalyst displays negligible activity decay, outperforming the benchmark Pt/C catalyst.

## Results

### Preparation of atomically thin PtTe_2_ NSs

**Fig. 1:**
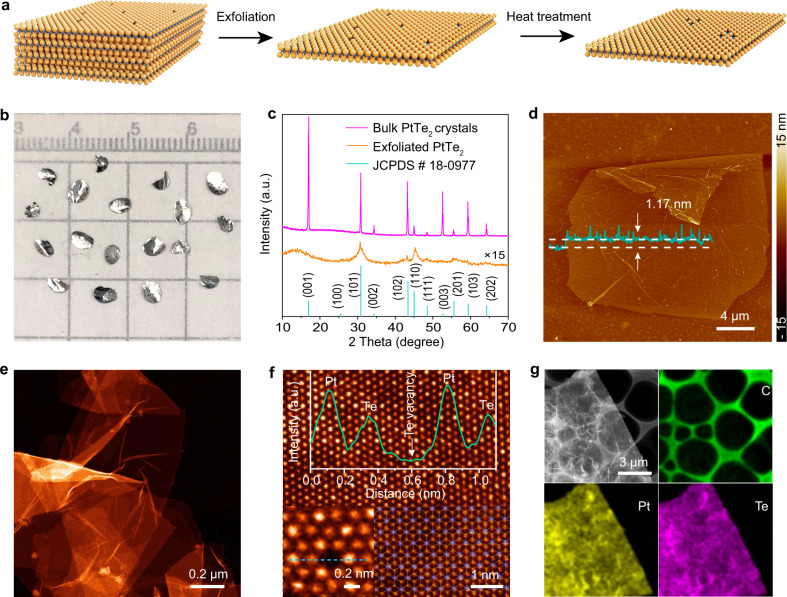
Preparation and characterization of PtTe_2_ NSs. **a** Schematic illustration showing the fabrication of atomically thin PtTe_2_ NSs with different vacancy structures. **b** Digital photograph of the PtTe_2_ crystals on a millimeter-grade paper. **c** XRD patterns of bulk PtTe_2_ crystals and exfoliated PtTe_2_ NSs. The standard XRD pattern of PtTe_2_ (JCPDS # 18-0977) is shown as a reference. **d** AFM image of atomically thin PtTe_2_ NSs. **e** HAADF-STEM image of few-layer PtTe_2_ NSs. **f** High-resolution HAADF-STEM image of few-layer PtTe_2_ NSs and the corresponding structural model of PtTe_2_. Inset on top shows the intensity profile corresponding to the blue line. **g** HAADF-STEM image and the corresponding elemental mapping images of exfoliated PtTe_2_ NSs.

PtTe_2_ crystallizes in a CdI_2_-type trigonal (1 T) structure (P$\bar{3}$m1, a = b = 4.026 Å, c = 5.221 Å) with adjacent layers (interlamellar space: 3.52 Å) connected via weak Van der Waals interaction (Supplementary Fig. 1)^17^. In this work, a chemical vapor transport (CVT) technique was employed to synthesize bulk PtTe_2_ crystals with closely stacked lamellar architecture (Supplementary Fig. 2), following by electrochemical exfoliation (detailed in Supplementary Figs. 3 and 4) to produce atomically thin 2D PtTe_2_ NSs as illustrated in Fig. 1a. Notably, a wealth of Te-SAVs was generated during the crystal growth period (discussed in the following part). The exfoliated PtTe_2_ NSs display broad (101), (102), (110), and (201) diffraction peaks in the X-ray diffraction (XRD) pattern (Fig. 1c). The disappearance of strong and sharp diffraction peaks results from loss of long-range order in PtTe_2_ after exfoliation^18,19^. Atomic force microscopy (AFM) measurements show that the PtTe_2_ NSs have lateral dimensions of ~1–15 μm with thicknesses of ~0.6–6 nm (average thickness: ~3 nm) (Fig. 1d and Supplementary Fig. 5). To examine the morphology, atomic structure, and chemical composition of the exfoliated PtTe_2_ NSs, microscopy characterizations including transmission electron microscopy (TEM), and aberration-corrected high-angle annular dark-field scanning TEM (HAADF-STEM) were performed. Figure 1e displays a low magnification HAADF-STEM image, showing the corrugated and roughened 2D surface of few-layer PtTe_2_ NSs. Notably, the high-resolution HAADF-STEM image (Fig. 1f), viewed from the [001] zone axis, displays atomic lattice composing of alternating bright and dark spots, which correspond, respectively, to the Pt and Te atomic columns as indicated in the atomic model (inset at the lower right corner of Fig. 1f). Furthermore, vacancies can be directly visualized with distinguishable contrast in the few-layer PtTe_2_ NSs (inset at the lower left corner of Fig. 1f). The corresponding line intensity profile (inset in the upper portion of Fig. 1f) combined with the atomic model (inset at the lower right corner of Fig. 1f) indicates that the point defect corresponds to Te vacancy. Additionally, the elemental mapping shows a homogenous distribution of Pt and Te elements over the whole PtTe_2_ NSs (Fig. 1g). No carbon signal is detected on the surface of exfoliated PtTe_2_ NSs, indicating intercalator and/or its decomposition products can be washed away after exfoliation. Such a conclusion can be further supported by the elemental analysis result, which reveals that the content of carbon in the exfoliated PtTe_2_ NSs is about zero.

### Thermal induced migration of Te-SAVs in PtTe_2_ NSs

Prior to the heat treatment, the thermal stability of PtTe_2_ NSs was investigated by thermogravimetry analysis (TGA) at ambient pressure, and the results (Supplementary Fig. 6) indicate high thermal stability of PtTe_2_ NSs up to ~650 °C. Accordingly, PtTe_2_ NSs were heated to 200, 400, and 600 °C, respectively, in Ar (gas flowrate: 200 sccm) for 1 h, yielding PtTe_2_-200 NSs, PtTe_2_-400 NSs, and PtTe_2_-600 NSs. Intriguingly, based on the result of inductively coupled plasma-optical emission spectroscopy (ICP–OES; Perkin Elmer Avio 500, UK), the PtTe_2_ NSs keep a constant atomic ratio of Pt/Te during the entire heat treatment process (Supplementary Table 1, Supplementary Fig. 7). Additionally, other characterizations including XRD (Supplementary Fig. 8), TEM (Supplementary Figs. 9–11), and X-ray photoelectron spectroscopy (XPS, Supplementary Fig. 12) all show negligible changes in morphology and composition of PtTe_2_ NSs during heat treatment.

**Fig. 2:**
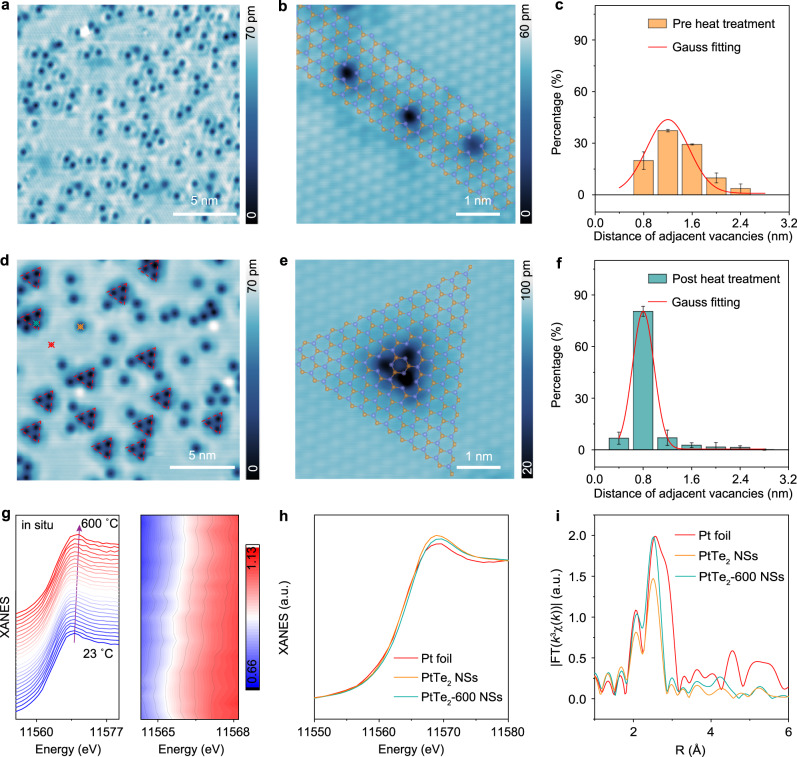
Heat treatment-induced migration of Te-SAVs in PtTe_2_. **a** Large-scale STM image of thin PtTe_2_ sheets taken at *V*_S_ = −2.0 V and *I* = 30 pA. **b** Close-up STM image showing three individual Te-SAVs on the surface of thin PtTe_2_ sheets with the corresponding structural model superimposed. The yellow and blue atoms represent Te and Pt atoms, respectively. Te atoms at the bottom of PtTe_2_ structural model are omitted. **c** Statistic distance of neighboring Te-SAVs in thin PtTe_2_ sheets before heat treatment. **d** Large-scale STM image of thin PtTe_2_ sheets after heat treatment. **e** Close-up STM image showing the formation of ordered trigonal Te-SAV clusters on the surface of thin PtTe_2_ sheets after heat treatment with the corresponding structural model superimposed. Te atoms at the bottom of PtTe_2_ structural model are omitted. **f** Statistic distance of neighboring Te-SAVs in thin PtTe_2_ sheets after heat treatment. The error bars in **c** and **f** represent standard deviation of different technical replicates. **g** Stacking plots of the in situ Pt L_3_-edge XANES spectra of PtTe_2_ NSs collected from room temperature to 600 °C in He gas (left), and the corresponding 2D contour plots of the in situ Pt L_3_-edge XANES spectra as shown in **g** (right), revealing the positive shift of the white line. **h** Normalized Pt L_3_-edge XANES spectra for PtTe_2_ NSs, PtTe_2_-600 NSs, and Pt foil. **i** Fourier-transformed *k*^3^-weighted EXAFS spectra for PtTe_2_ NSs, PtTe_2_-600 NSs, and Pt foil.

To show heat treatment-induced migration of Te-SAVs in PtTe_2_ NSs, scanning tunneling microscopy (STM) was performed to better visualize the evolution of Te-SAVs as a function of annealing temperature. Prior to heat treatment, as shown in Fig. 2a, a high density of uniformly dispersed SAVs (black dots) are observed on the surface of thin PtTe_2_ sheets. Figure 2b displays the atomically resolved STM image of three individual Te-SAVs on the surface. A superimposition of the atomic structure over the close-up STM image reveals that atomic vacancies reside at the Te sites. This conclusion can be further supported by ICP–OES and HAADF-STEM results mentioned above as well as other reported transition metal dichalcogenide systems^20,21^, demonstrating the vacancies are single atomic Te vacancies. In addition, statistical analysis of STM results in Fig. 2c shows that all Te-SAVs are separated by a certain distance from about 0.8 to about 2.4 nm. Fascinatingly, the heat treatment process ubiquitously induces migration of Te-SAVs in PtTe_2_ to form ordered trigonal Te-SAV clusters on the surface, as highlighted by red triangle dotted box in Fig. 2d. In addition, these trigonal Te-SAV clusters become dominant after heat treatment. By superimposing the atomic model on the close-up STM image in Fig. 2e, it can be seen that the cluster consists of three Te-SAVs, each of which is still separated by a Te atom. Figure 2f analyzes the distance between adjacent Te-SAVs after heat treatment. As expected, the distance of two neighboring Te-SAVs decreases significantly after forming clusters. Thus, different from traditional works that mainly focus on regulating the number of vacancies and doping vacancies via heteroatoms^22^, the obtained PtTe_2_ exhibits a well-defined atomic structure, which can be served as a model catalyst to investigate the influence of vacancy evolution on the catalytic performance.

The chemical states of PtTe_2_ were examined by XPS. As shown in Supplementary Fig. 12, for bulk PtTe_2_ crystals, the core-level Pt 4 *f* XPS spectrum exhibits two peaks at binding energies of 71.1 (4*f*_7/2_) and 74.4 (4*f*_5/2_) eV, which can be assigned to metallic Pt^23^. Two zero valent state peaks associated to Te^0^ at 572.0 (3*d*_5/2_) and 582.3 eV (3*d*_3/2_), together with two oxidation state peaks associated to Te^IV^ arising from the Te = O bonds after exposing PtTe_2_ crystals in air, are detected in Te 3*d* XPS spectrum^23^. After exfoliation and heat treatment, both Pt and Te in PtTe_2_ NSs, PtTe_2_-200 NSs, PtTe_2_-400 NSs, and PtTe_2_-600 NSs still mainly exist in their metallic states, and the gradual appearance of positive binding energy shift of Pt 4*f*_7/2_, Pt 4*f*_5/2_, Te 3*d*_5/2_, and Te 3*d*_3/2_ with increasing heat treatment temperature may result from the influence of improved oxygen adsorption after air exposure or electron transfer between Pt and Te atoms. To avoid exposing the PtTe_2_ NSs to air and therefore provide a definitive study of the electronic structure and coordination environment of Pt in exfoliated PtTe_2_ NSs during thermal treatment, in situ X-ray absorption near-edge structure (XANES) spectra of Pt L_3_ edge were collected in a home-made testing setup from room temperature to 600 °C in He gas^24^. The results (Fig. 2g) show that with increasing heat treatment temperature, a slight positive shift of Pt L_3_ edge of XANES is observed, indicating a slight increase in average valance state of Pt in PtTe_2_ NSs during Te-SAVs migration. After cooling down to room temperature in He, the Pt L_3_ edge XANES spectrum of PtTe_2_-600 NSs was collected and compared with pristine PtTe_2_ NSs, and Pt foil. As revealed in Fig. 2h, the edge energy of pristine PtTe_2_ NSs is the same as that of Pt foil, suggesting that the Pt atoms in PtTe_2_ NSs are mainly present in zero valance state, which is in accordance with the XPS results. However, the Pt L_3_ absorption edge of PtTe_2_-600 NSs shows a slight positive shift compared with that of the pristine PtTe_2_ NSs, revealing that clustering of Te-SAVs in PtTe_2_ NSs would lead to a decrease in the number of Pt electrons. Additionally, L_3_-edge XANES spectra of Te in PtTe_2_ samples were collected and compared in Supplementary Fig. 13a. As expected, the Te absorption edge position of PtTe_2_-600 NSs is lower in energy than that of PtTe_2_ NSs, indicating decreased average valance state of Te in PtTe_2_-600 NSs. The above results indicate electron transfer from Pt to Te atoms in PtTe_2_ NSs during thermal treatment. In addition, both PtTe_2_ NSs and PtTe_2_-600 NSs show higher white line intensities of the Pt L_3_-edge as compared to Pt foil, suggesting a higher density of state of *d* band vacancy for Pt atoms in PtTe_2_ than that for metallic Pt^25^. Figure 2i shows the corresponding Fourier-transformed (FT) *k*^3^-weighted extended X-ray absorption fine structure (EXAFS) spectrum for PtTe_2_ NSs, PtTe_2_-600 NSs, and Pt foil. It can be seen that the EXAFS spectrum for PtTe_2_ NSs and PtTe_2_-600 NSs display similar coordination environment except that the amplitude of the first shell peak (at 2.51 Å) for PtTe_2_-600 NSs is obviously enhanced, which could be due to the decreased local-structure disorder around Pt atom after migration of Te-SAVs to form Te-SAV clusters. Such a phenomenon can be further displayed by the enhanced amplitude from PtTe_2_ NSs to PtTe_2_-600 NSs in the EXAFS curves in *K* space (Supplementary Fig. 13b), and the results of EXAFS fitting, where the disorder degree of PtTe_2_-600 NSs is 0.0028 ± 0.0004, much lower than that of PtTe_2_ NSs (0.0037 ± 0.0007).

### Properties of PtTe_2_ in HER

**Fig. 3:**
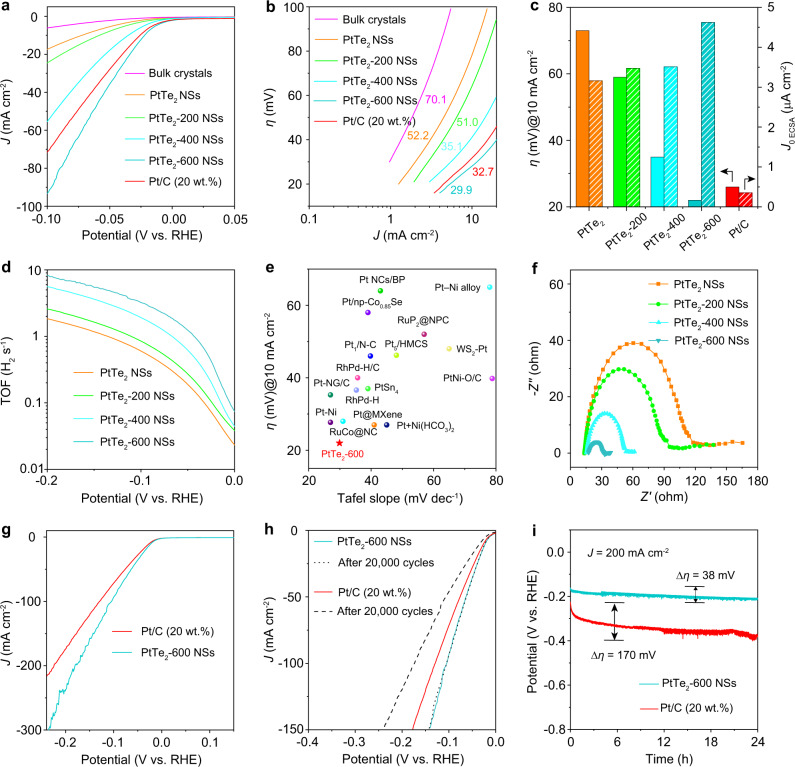
HER performance. **a** LSV curves of bulk PtTe_2_ crystals, PtTe_2_ NSs, PtTe_2_-200 NSs, PtTe_2_-400 NSs, PtTe_2_-600 NSs, and Pt/C recorded in 1.0 M KOH at a scan rate of 5 mV s^−1^, with 80 % iR compensation. **b** The corresponding Tafel plots. **c** Comparison of overpotential at 10 mA cm^−2^ and *J*_0 ECSA_. **d** The potential-dependent TOF curves for PtTe_2_ NSs, PtTe_2_-200 NSs, PtTe_2_-400 NSs, and PtTe_2_-600 NSs. **e** Performance comparison of PtTe_2_-600 NSs with other reported noble metal-based HER catalysts with high activity in 1.0 M KOH. The detailed information of these catalysts was listed in Supplementary Table 2. **f** Nyquist plots collected at an overpotential of 100 mV. **g** Comparison of LSV curves for PtTe_2_-600 NSs and Pt/C at large current density. **h** LSV curves of PtTe_2_-600 NSs and Pt/C before and after 20,000 cycles of accelerated CV test. **i** Chronopotentiometry measurement recorded at 200 mA cm^−2^ for 24 h without iR compensation.

The electrocatalytic performances of various PtTe_2_ samples were evaluated in both acidic and alkaline electrolyte. As displayed in the linear sweep voltammetry (LSV) curves in Fig. 3a, PtTe_2_ NSs show substantially larger geometric HER current density (*J*) than bulk PtTe_2_ crystals at the same overpotential resulting from the more exposed active sites of PtTe_2_ NSs after exfoliation^19,26^. Interestingly, the HER activity is gradually enhanced from PtTe_2_ NSs to PtTe_2_-600 NSs because of the accelerated catalytic kinetics as shown in the corresponding Tafel slopes (Fig. 3b, Supplementary Fig. 14)^27^. The PtTe_2_-600 NSs feature an exceptionally low HER onset potential (~0 mV, Supplementary Fig. 15) and overpotential (22 mV at *η* = 10 mA cm^−2^, left of Fig. 3c), superior to the state-of-the-art 20 wt.% Pt/C catalyst (26 mV at *η* = 10 mA cm^−2^, left of Fig. 3c). To further display the much-enhanced catalytic activity, the exchange current density (*J*_0_) based on electrochemically active surface area (ECSA) was calculated by extrapolating the Tafel plot (Supplementary Fig. 14)^28–30^. It shows that *J*_0 ECSA_ increases in the order of PtTe_2_ NSs (3.16 μA cm^−2^) < PtTe_2_-200 NSs (3.46 μA cm^−2^) < PtTe_2_-400 NSs (3.51 μA cm^−2^) < PtTe_2_-600 NSs (4.62 μA cm^−2^), while *J*_0 ECSA_ of Pt/C is 0.35 μA cm^−2^ (right of Fig. 3c). Additionally, the TOF trend (measured at −0.2 V vs. RHE) also follows the same order with PtTe_2_ NSs (1.84 s^−1^) < PtTe_2_-200 NSs (2.59 s^−1^) < PtTe_2_-400 NSs (5.59 s^−1^) < PtTe_2_-600 NSs (8.21 s^−1^) (Fig. 3d). Considering that PtTe_2_-600 NSs show similar composition with PtTe_2_ NSs (ICP–OES results), PtTe_2_-600 NSs exhibit much-enhanced intrinsic HER catalytic activity, outperforming most of the reported highly active HER catalysts (Fig. 3e, and Supplementary Table 2). The high HER activity of the PtTe_2_-600 NSs can be further supported by the electrochemical impedance spectroscopy (EIS) results (Fig. 3f, Supplementary Fig. 16), which display much reduced charge transfer resistance compared to the PtTe_2_ NSs^31^. Even at large current densities, the PtTe_2_-600 NSs still display superior HER activity than commercial Pt/C (Fig. 3g). The LSV curves based on mass activity of Pt in PtTe_2_-600 NSs and Pt/C are shown in Supplementary Fig. 17. The mass activity of Pt in PtTe_2_-600 NSs is obviously higher than that in Pt/C at the same potential. At −0.2 V vs. RHE, the mass activity of Pt in PtTe_2_-600 NSs is calculated to be 1.55 A mg_Pt_^−1^, while that is only 1.13 A mg_Pt_^−1^ for Pt in Pt/C. Apart from the activity, catalytic stability is equally important in practical applications. Accelerated cyclic voltammetry (CV) test elucidates the robustness of PtTe_2_-600 NSs in HER catalysis with virtually unchanged polarization curves even after 20,000 CV cycles (Fig. 3h). Furthermore, long-term chronopotentiometry measurement shows a slight change of overpotential after 24 h at a constant current density of 200 mA cm^−2^ for PtTe_2_-600 NSs, much better than that of Pt/C catalyst (Fig. 3i). The PtTe_2_-600 NSs after long-term stability test were characterized, showing negligible change of morphology, structure, and composition (Supplementary Fig. 18). The similar catalytic trend of PtTe_2_ for HER was also demonstrated in the acidic electrolyte, as shown in Supplementary Fig. 19.

### Theoretical insights between vacancy clustering and HER

To shed light on the influence of ordered clustering of Te-SAVs in atomically thin PtTe_2_ on HER activity, a series of theoretical calculations were performed based on the first principle methods. When the thickness increases from monolayer to bilayer, PtTe_2_ evolves from semiconductive (bandgap: ~0.84 eV) to metallic. The electronic structure of trilayer PtTe_2_ has already resembled to its bulk material (Supplementary Figs. 20 and 21). Since metallic nature is of pivotal importance for hydrogen adsorption, the bilayer PtTe_2_ is expected to show good HER activity, which can be confirmed by calculating the hydrogen adsorption energy at active sites in different layered PtTe_2_ (Supplementary Fig. 22). To reduce the computational cost, the metallic bilayer PtTe_2_ was adopted as the theoretical model for the following calculations.

**Fig. 4:**
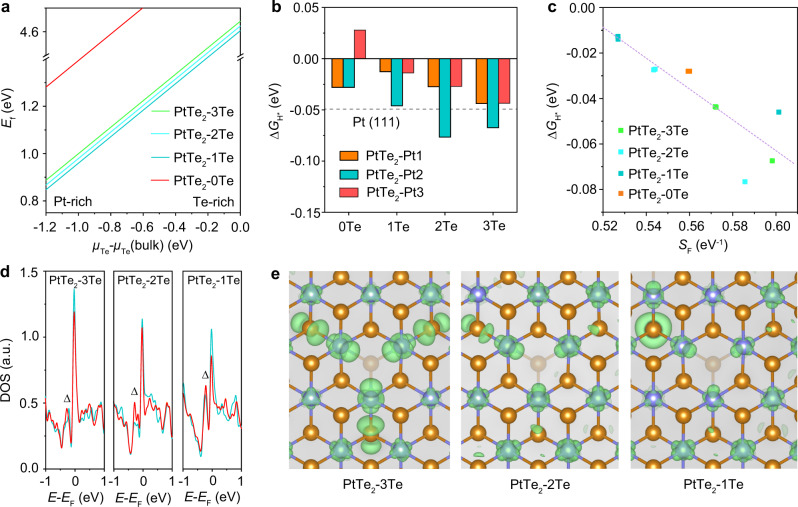
Electronic structure and binding strength of H. **a** Formation energy of bilayer PtTe_2_-3Te, PtTe_2_-2Te, PtTe_2_-1Te, and PtTe_2_-0Te structures. **b** The calculated hydrogen adsorption free energy of different undercoordinated Pt sites in PtTe_2_. **c** Dependence of hydrogen adsorption free energy on Fermi softness of Pt sites in various PtTe_2_ structures. The nominal electronic temperature in weight function was set to 0.1. **d** DOS curves of Pt sites in PtTe_2_-3Te, PtTe_2_-2Te, and PtTe_2_-1Te structures. The orange, blue, and red lines represent the DOS of Pt1, Pt2, and Pt3 sites in the corresponding PtTe_2_-XTe structure, respectively. **e** The corresponding partial charge. The isosurface level was set to 0.002 e Å^−3^.

To uncover the dependence of clustering degree of Te vacancies on the heat treatment temperature, the relative stability of bilayer PtTe_2_ with different types of Te vacancies was investigated by calculating their formation energy (*E*_f_)^32^. As displayed in Supplementary Fig. 23, the bilayer PtTe_2_ models, containing three Te vacancies and separated by different numbers of Te atoms, were constructed to model the different structures of PtTe_2_ NSs. For simplicity, herein, we use PtTe_2_-xTe (x = 3, 2, 1, or 0), where “x” refers to the number of Te atoms between two isolated Te vacancies on the surface of bilayer PtTe_2_, to denote the structure. Specifically, the PtTe_2_-3Te represents the exfoliated PtTe_2_ NSs with isolated Te-SAVs, the PtTe_2_-1Te represents the PtTe_2_-600 NSs with plenty of trigonal Te-SAVs, while the PtTe_2_-2Te represents the intermediate transition state of PtTe_2_ NSs during heat treatment, where the distance between two Te-SAVs is closer than that of Te-SAVs in PtTe_2_ NSs. Figure 4a shows the calculated *E*_f_ based on the energy difference between the intact PtTe_2_ and defective PtTe_2_-xTe. The *E*_f_ decreases in the order of PtTe_2_-1Te < PtTe_2_-2Te < PtTe_2_-3Te ≪ PtTe_2_-0Te, suggesting that heat treatment would lead to formation of PtTe_2_-1Te because of the lowest *E*_f_, which explains the formation of ordered trigonal Te-SAV clusters in PtTe_2_-600 NSs (Fig. 2e)_._ In addition, when Te-SAVs coalesce into a larger vacancy, the *E*_f_ greatly increases, indicating the structure of PtTe_2_-0Te hardly exists in the sample. This unstable structure may be ascribed to the formation of lower coordinated Pt atoms.

The step of H_2_O dissociation is considered in alkaline solution, which may exert extra barrier on the HER process^33^. As shown in Supplementary Fig. 24, the barrier of H_2_O dissociation on defective PtTe_2_ (1.09 eV) is very close to that on Pt (111) surface (1.07 eV), indicating that H_2_O dissociation has a negligible impact on the activity difference between PtTe_2_ and Pt for HER. The HER performance of PtTe_2_-xTe was then evaluated by Δ*G*_H*_, where a higher Δ*G*_H*_ indicates a weaker hydrogen adsorption, and vice versa. An ideal HER catalyst should bind to hydrogen neither too strongly nor too weakly, giving rise to Δ*G*_H*_ close to zero^34^. The calculated hydrogen adsorption energies reveal that the undercoordinated Pt sites rather than Te sites in PtTe_2_-1Te are the active sites (Supplementary Fig. 25), which was further verified by the poison experiments (Supplementary Fig. 26). Subsequently, the location of different undercoordinated Pt sites was investigated, denoted as PtTe_2_-Pty (y = 1, 2, or 3), where “y” is the specific atomic position of Pt atoms (Supplementary Fig. 23). Figure 4b shows the calculated Δ*G*_H*_ on different undercoordinated Pt sites in PtTe_2_-xTe and the Pt site with the lowest | Δ*G*_H*_ | was chosen as the dominate active site of the corresponding catalysts. It can be observed that the Δ*G*_H*_ for PtTe_2_-xTe varies in the following order: PtTe_2_-3Te (−0.044 eV) < PtTe_2_-2Te (-0.027 eV) < PtTe_2_-1Te (−0.013 eV) > PtTe_2_-0Te (−0.028 eV). In addition, the Pt1 and Pt3 sites in PtTe_2_-Pty possess a significantly lower value of Δ*G*_H*_ as compared to the Pt2 site. To experimentally verify the gradually increased Δ*G*_H*_ of Pt sites in PtTe_2_, CV measurements of PtTe_2_ NS, PtTe_2_-200 NS, PtTe_2_-600 NS, and Pt/C were performed in Ar purged 1.0 M KOH. As shown in Supplementary Fig. 27, the underpotentially deposited hydrogen (H_upd_) peak of PtTe_2_-600 NSs at ~0.21 V vs. RHE is negatively shifted relative to that of PtTe_2_ NSs (~0.26 V vs. RHE) and PtTe_2_-200 NSs (~0.25 V vs. RHE), suggesting weaker adsorption of hydrogen on PtTe_2_-600 NSs^35,36^. Additionally, the H_upd_ peak of PtTe_2_-600 NSs is more negative than that of the Pt/C catalyst. From these mutually corroborating data (STM, *E*_f_, Δ*G*_H*_, and CV), it can be concluded that during heat treatment, the Te-SAVs in atomically thin PtTe_2_ NSs migrate to form ordered Te-SAV clusters, which effectively reduces the adsorption strength of hydrogen on the Pt sites and thus drastically enhances the hydrogen evolution performance.

Last but not the least, the Fermi softness, defined by Eq. (1), was established to highlight the underlying origin between Δ*G*_H*_ and the electronic structure of PtTe_2_.

1 $${S}_{{\rm{F}}}=\int g\left({\rm{E}}\right)w\left({\rm{E}}\right){\rm{dE}}$$

Herein, $g\left({\rm{E}}\right)$ and $w\left({\rm{E}}\right)$ denote the density of states (DOS) and weight function, respectively^37^$,w\left({\rm{E}}\right)$ is assigned by the derivative of Fermi-Dirac distribution function at non-zero temperature. As revealed in Fig. 4c, by including all Pt sites in different PtTe_2_-xTe, an obvious linear relationship between Δ*G*_H*_ and ${S}_{{\rm{F}}}$ can be established. It is noteworthy that the PtTe_2_-1Te, bearing a smaller *S*_F_, features reduced hydrogen adsorption, suggesting that the DOS near the Fermi energy (*E*_F_) of Pt sites greatly influences the Δ*G*_H*_. The changes in the DOS of Pt sites were further validated in Fig. 4d and Supplementary Fig. 28, where the peak of DOS near the *E*_F_ displays a decreased order: PtTe_2_-3Te > PtTe_2_-2Te > PtTe_2_-1Te, indicating a decreased DOS of Pt sites after the clustering of regular Te-SAVs. In addition, the total DOS of the Pt and Te atoms around the Te defects in PtTe_2_-3Te, PtTe_2_-2Te, and PtTe_2_-1Te structure (Supplementary Fig. 29a-b) shows a similar variation trend. To verify this theoretical finding, we performed scanning tunneling spectroscopy (STS, d*I*/d*V* vs. V) to probe the local DOS over different vacancy structures. The results show that near-Fermi local DOS of defect-free site, Te-SAV and trigonal Te-SAV in PtTe_2_ (marked to red, orange, and blue symbols, respectively, in Fig. 2d) gradually decrease (Supplementary Fig. 29c). The reduced DOS of Pt sites near *E*_F_ may result from two possible factors: (1) Electron transfer from Pt to Te atoms. As demonstrated in the in situ XANES results, the normalized Pt L_3_-edge XANES spectra display a slight positive shift, while the Te absorption edge of PtTe_2_-600 NSs is lower in energy, compared with that of the pristine PtTe_2_ NSs. (2) The interaction between neighboring Te-SAVs as evidenced by the increased intensity of the DOS peak adjacent to the *E*_F_ (noted with ‘Δ’ in Fig. 4d). In addition, partial charge density in the vicinity of *E*_F_ was calculated to better visualize the decreased electronic states of Pt sites (Fig. 4e and Supplementary Fig. 30). The results confirm that all undercoordinated Pt sites possess interacting orbital directing to the center of Te vacancy, and the orbital volume gradually decreases as the neighboring Te-SAVs approach to each other. In conclusion, as both reduced DOS and deceased interacting orbital volume of Pt sites are detrimental to hydrogen adsorption, the ordered clustering of Te-SAVs in atomically thin PtTe_2_ NSs effectively reduces hydrogen adsorption, rendering them highly active in HER.

## Discussion

In conclusion, we have developed a facile exfoliation followed by thermal annealing approach to engineer the defects in atomically thin PtTe_2_ NSs as a model catalyst to understand the correlation between electronic structure, adsorption energy, and hydrogen evolution activity of atomically defined Pt sites. The Te-SAVs in the exfoliated PtTe_2_ NSs migrate to form ordered trigonal Te-SAV clusters during heat treatment, which effectively reduces the adsorption energy of hydrogen and promotes the kinetics of HER. In addition, benefiting from the exposed undercoordinated Pt sites with robust stability in atomically thin PtTe_2_, the resulting catalysts exhibit superior activity and stability for HER, compared with commercial Pt/C catalysts. The finding here provides a strategy to engineer geometrically well-defined active sites via the clustering of atomic defects, which allows for the understanding of the correlations between electronic structure of catalytic center and catalytic performance.

## Method

### Materials

Platinum (99.98%), tellurium (99.999%) were purchased from Alfa-Aesar and stored in the glove box. Tetrabutylammonium tetrafluoroborate (TBAB), dimethyl sulfoxide (DMSO), potassium hydroxide (KOH), and Nafion solution (5 wt.%) were purchased from Sigma–Aldrich, Pt/C (20 wt.%) catalysts were purchased from Alfa Aesar, and pure Argon gas (Purity: 99.999 %), pure hydrogen gas (Purity: 99.999 %) were purchased from Chem-Gas Pte Ltd (Singapore). All chemical reagents were utilized as received without further purification. Water was purified through a Millipore system.

### Synthesis of bulk PtTe_2_ crystals

Bulk PtTe_2_ crystals were synthesized via a CVT method in a three-zone tube furnace (OTF-1200X-III-S-UL, MTI Corporation, USA). First, 1 g of high-purity Pt (0.433 g) and Te (0.567 g) powders with a stoichiometric molar ratio of 1: 2 were ground thoroughly in a glove box. Then, the mixed powders were sealed in an evacuated quartz tube (length: 10 cm; external diameter: 13 mm; and wall thickness: 1 mm) under vacuum using an oxygen/hydrogen welding torch. Next, the sealed tube was placed in the furnace and heated to 1000 °C for 48 h. Afterwards, the temperature was further increased to 1150 °C for another 1 h. Finally, the furnace was slowly cooled to ambient temperature, and the bulk PtTe_2_ crystals were collected.

### Electrochemical exfoliation of bulk PtTe_2_ crystals

Bulk PtTe_2_ crystals were electrochemically exfoliated into atomically thin PtTe_2_ NSs using an electrochemical workstation (CH Instruments, Inc., USA) consisting of a three-electrode system. Before exfoliation, the bulk PtTe_2_ crystals were sliced into thin specimens and served as the working cathode, a Pt wire electrode was used as the counter electrode, and 0.05 M TBAB-DMSO solution was used as the electrolyte. The exfoliation of PtTe_2_ crystals was performed by applying a bias of −5.0 V on the working electrode. After exfoliation, the obtained product was collected by centrifugation, washed with plenty of H_2_O and ethanol, and dried in a vacuum oven.

### Thermal treatment of PtTe_2_ NSs

The exfoliated PtTe_2_ NSs were placed in a quartz boat and heated in a tube furnace to T (T = 200, 400, or 600 °C) at 5 °C min^−1^ in argon gas (flowrate: 200 sccm) for 1 h. After cooling to room temperature, the PtTe_2_-T NSs were obtained.

### Electrochemical measurements

The electrochemical measurements were performed on a standard three-electrode system using a rotating disk electrode setup (WaveVortex 10, USA) at room temperature (22 °C). A catalyst coated glassy carbon electrode (geometric area: 0.19625 cm^2^), a carbon rod and a Hg/HgCl electrode (KCl-saturated) were used as the working, counter, and reference electrode, respectively. Argon saturated 1.0 M KOH or 0.5 M H_2_SO_4_ solution was used as the electrolyte. Before loading catalysts, glassy carbon electrode (GCE) was successively polished with 1.0, 0.3, and 0.05 mm Al_2_O_3_ slurry to obtain an ultraclean surface. Afterwards, 4 mg of catalysts were dispersed in 1 ml of ethanol with 8 μl of Nafion 117 solution and sonicated for ~2 min to obtain a homogeneous catalyst ink. For commercial Pt/C (20 wt.%), 40 μl of the catalyst ink was dropped onto the GCE and allowed to dry at room temperature. Thus, the average loading of the catalyst is 0.809 mg cm^−2^, and the total mass loading of Pt is 0.162 mg cm^−2^. For PtTe_2_ (n_Pt_: n_T__e_ = 0.61), 16.6 μl of the catalyst ink was dropped onto the GCE and dried at room temperature. The average loading of the catalyst is 0.336 mg cm^−2^, and the Pt content is ~0.162 mg cm^−2^, which is the same as that in Pt/C (20 wt.%) catalysts. To investigate the influence of Pt/C (20 wt.%) catalyst loading amount on HER, the catalyst loading-dependent current density at a particular overpotential was studied. As shown in Supplementary Fig. 31, when the loading amount of Pt/C increases from 0.4 mg cm^−2^ to 0.9 mg cm^−2^, the current density almost increases linearly (blue dash line). At this stage, the number of active sites dominate the HER process, rather than other factors (e.g., mass transport, conductivity). At the loading amount of Pt/C of 1.0 mg cm^−2^, the current density starts to decrease sharply.

All LSV curves were collected at a rotation speed of 1600 rpm and a sweep rate of 5.0 mV s^−1^ with 80 % of ohmic drop compensation. The reference electrode was calibrated in a H_2_-saturated 1.0 M KOH and 0.5 M H_2_SO_4_ solution at a scan rate of 5.0 mV s^−1^ (Supplementary Fig. 32)^15,34,38^. EIS measurements were conducted at an overpotential of 100 mV in the frequency range of 0.01 to 10^5^ Hz with an amplitude of 5 mV. The accelerated stability test was performed by measuring the LSV curves before and after 20,000 continuous CV cycles in the potential range from −0.2 to 0.0 V vs. RHE with a sweep rate of 200 mV s^−1^. The durability of the catalyst was evaluated by the chronopotentiometry method.

### ECSA calculation

The CV measurement was performed at potentials between 0 and 1.25 V vs. RHE at a scan rate of 50 mV s^−1^ in Argon-purged 1.0 M KOH solution at room temperature. ECSA of PtTe_2_ NSs, PtTe_2_-200 NSs, PtTe_2_-400 NSs, PtTe_2_-600 NSs, and Pt/C (20 wt.%) were then calculated based on the reported method and Eq. (2)^28–30^:

2 $${\rm{ECSA}}=\frac{{Q}_{{\rm{H}}}}{0.21\times [{\rm{Pt}}]}=\frac{{S}_{{\rm{area}}}}{{V}_{{\rm{scan}}}\times 0.21\times [{\rm{Pt}}]}$$

where *Q*_H_ (mC) is the average charge integrated from hydrogen adsorption/desorption process (0.0–0.5 V vs. RHE) on the CV curve, which can be determined by the ratio of integrating hydrogen adsorption/desorption peak area with the subtraction of the double layer to scanning velocity (0.05 V s^−1^). 0.21 mC cm^−2^ is the electrical charge associated with monolayer adsorption of hydrogen on Pt, and [Pt] is the loading of Pt on the working electrode (0.0318 mg in our experiments).

### TOF calculation

To calculate the TOF per undercoordinated Pt sites in the PtTe_2_ catalyst, we used the following Eq. (3):

3 $${\rm{TOF}}=\frac{\#\,{\rm{Total}}\,{\rm{hydrogen}}\,{\rm{turnover}}\,{\rm{per}}\,{\rm{geometric}}\,{\rm{aera}}}{\#\,{\rm{Active}}\,{\rm{sites}}\,{\rm{per}}\,{\rm{geometrica}}\,{\rm{area}}}$$

The total number of hydrogen turn overs was calculated from the current density according to the Eq. (4)^39,40^:

4 $${\rm{\#Totalhydrogenturnovers}} 	=\left(J\frac{{\rm{mA}}}{{{\rm{cm}}}^{2}}\right)\left(\frac{1{\rm{C}}/{\rm{s}}}{1000{\rm{mA}}}\right)\left(\frac{1{\rm{mol}}{{\rm{e}}}^{-}}{96485.3{\rm{C}}}\right)\left(\frac{1{\rm{mol}}{{\rm{H}}}_{2}}{2{\rm{mol}}{{\rm{e}}}^{-}}\right)\left(\frac{6.02\times {10}^{23}{\rm{molecules}}{{\rm{H}}}_{2}}{1{\rm{mol}}{{\rm{H}}}_{2}}\right)\\ 	 =3.12\times {10}^{15}\frac{{{\rm{H}}}_{2}/{\rm{s}}}{{{\rm{cm}}}^{2}}{\rm{per}}\frac{{\rm{mA}}}{{{\rm{cm}}}^{2}}$$

The upper limit number of active sites was calculated based on the hypothesis that all defective Pt sites in the PtTe_2_ catalysts formed active centers and all of them were accessible to the electrolyte. The real number of active and accessible undercoordinated Pt sites should be considerably lower than the calculated value. According to ICP–OES results that n_Pt_: n_Te_ is 0.61, thus, the percentage of defective Pt sites in all Pt sites was calculated to be 18%. Thus, the active sites per geometrical area were obtained according to the Eq. (5):

5 $$	\#{\rm{Activesites}}({\rm{Pt}})\\ 	 \qquad= \left(\frac{{\rm{defective}}\,{\rm{Pt}}\,{\rm{wt}}. \% \times {\rm{catalyst}}\,{\rm{loading}}\big(\frac{{\rm{g}}}{{{\rm{cm}}}^{2}}\big)\,}{{\rm{Pt}}\,{\rm{Mw}}\,\big(\frac{{\rm{g}}}{{\rm{mol}}}\big)}\right)\left(\frac{6.022\times {10}^{23}}{1\,{\rm{mol}}\,{\rm{Pt}}}\right)\\ 	 \qquad=\left(\frac{48.25\, \% \times 18\, \% \times 0.3357\times {10}^{-3}\big(\frac{{\rm{g}}}{{{\rm{cm}}}^{2}}\big)}{195.05}\right)\left(\frac{6.022\times {10}^{23}}{1\,}\right)\\ 	 \qquad=9\times {10}^{16}\,{\rm{sites}}\,{{\rm{cm}}}^{-2}$$

Finally, the current density from the LSV polarization curve can be converted into TOF values according to the Eq. (6):

6 $${\rm{TOF}}=\frac{3.12\times {10}^{15}}{{9\times 10}^{16}}\times \left|J\right|=0.035\left|J\right|$$

### STM/STS measurement

The STM/STS measurements were conducted at T = 4.8 K in the CreaTec STM/AFM system under UHV. Before each measurement, the STM tip was calibrated on an Au(111) surface by checking the Shockley surface state. All the d*I*/d*V* spectra were taken through the standard lock-in technique with a modulation voltage of 10 mV and frequency of 713 Hz.

### Computational details

DFT calculations were carried out using the plane-wave technique with exchange-correlation interactions modeled by GGA-PBE functional^41^, as implemented in the Vienna ab initio Simulation package^42^. The ion–electron interactions were described by the projector augmented plane-wave approach^43^, and the cutoff energy was set to 400 eV. Structural optimizations were performed by minimizing the forces on all atoms to below 0.02 eV Å^−1^ and the energy to below 10^−5^ eV. Monkhorst-Pack method was adopted to sample the k-space with 7 × 7 × 5 mesh for PtTe_2_ unit-cell and 2 × 2 × 1 mesh for the surface of bilayer, respectively. The van der Waals correction was included using the Becke-Jonson damping with function parameters of the D2 method by Grimme et al.^43^. After fully relaxed, the lattice parameters of PtTe_2_ unit-cell were optimized to a = 4.01 Å and c = 5.00 Å, and the phonon spectrum (Supplementary Fig. 21) confirmed its dynamic stability in terms of no imaginary frequency. To explore the catalytic performance of layered PtTe_2_, a (8 × 8) slab with two layers along the (001) axis was constructed accompanying with a vacuum layer of 15 Å to avoid the interaction between neighboring images.

The free energy analysis method developed by Nørskov et al.^44^, was used to predict the reaction activity and the computational details can be referred to previous report^32^. We adopted Eq. (2): Δ*G*_H*_ = Δ*E*_H*_ + 0.24 eV (where Δ*E*_H*_ is the adsorption energy of H atom) to evaluate the adsorption free energy of H on different sites of PtTe_2_ surface. The formation energy of defective PtTe_2_ was calculated by the following Eq. (7):

7 $${E}_{{\rm{f}}}={E}^{{\rm{t}}}\left({\rm{def}}\right)-{E}^{{\rm{t}}}\left({\rm{ideal}}\right)+\sum {N}_{{\rm{Te}}}{\mu }_{{\rm{Te}}}$$

where ${E}^{{\rm{t}}}\left({\rm{def}}\right)$ and ${E}^{{\rm{t}}}\left({\rm{ideal}}\right)$ represent the total energy of defective and pristine PtTe_2_, and ${\mu }_{{\rm{Te}}}$ and ${N}_{{\rm{Te}}}$ represent the chemical potential of Te in PtTe_2_ and the vacancy number, respectively. Since it is impossible to obtain an accurate value of ${\mu }_{{\rm{Te}}}$, it varies between two limits, namely the ${\mu }_{{\rm{Te}}({\rm{bulk}})}$ and $1/2({E}_{{\rm{Pt}}{{\rm{Te}}}_{2}}-{\mu }_{{\rm{Pt}}({\rm{bulk}})})$.

## Supplementary information

Supplementary Information

Peer Review File

## Data Availability

The data supporting this study are available from the corresponding author upon reasonable request.
