# Supplementary material for: Ordered clustering of single atomic Te vacancies in atomically thin PtTe2 promotes hydrogen evolution catalysis
Source: Nat Commun. 2021 Apr 21;12:2351. doi: 10.1038/s41467-021-22681-4 (PMC8060321; doi:10.1038/s41467-021-22681-4)
Supplement: Supplementary file 1 — Supplementary Information [file 41467_2021_22681_MOESM1_ESM.docx]

Supplementary Information for

**Ordered Clustering of Single Atomic Te Vacancies in Atomically Thin PtTe_2_ Promotes Hydrogen Evolution Catalysis**

Li et al.

**
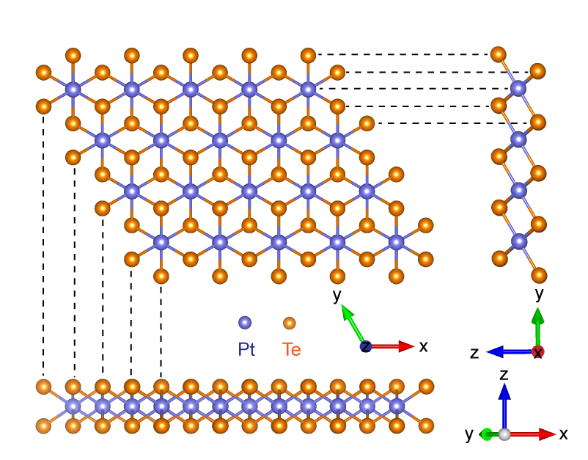
**

**Supplementary Figure 1.** Ball-and-stick model showing a monolayer of PtTe_2_ viewed from three different angles.

**
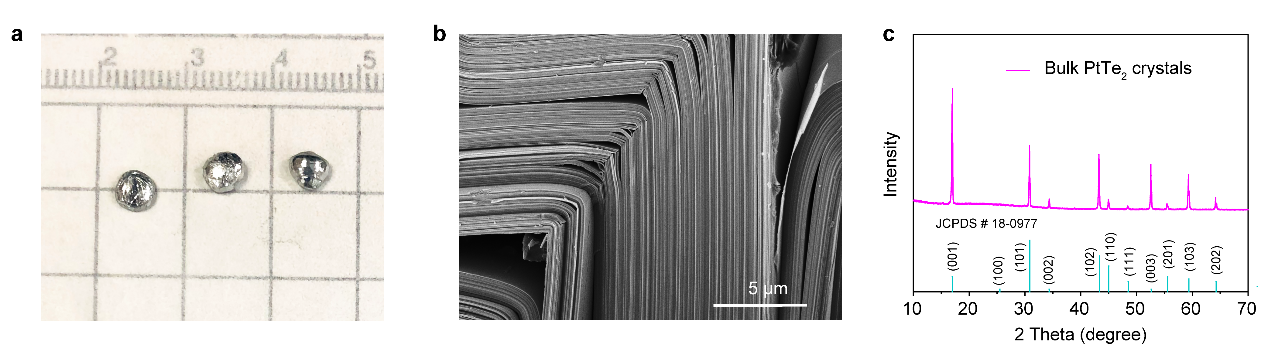
**

**Supplementary Figure 2.** **CVT method to prepare bulk PtTe_2_ crystals.** **a**, Photograph of the prepared bulk PtTe_2_ ingot. **b**, SEM image of the bulk PtTe_2_ crystals, revealing closely stacked lamellar architecture. **c**, XRD pattern of the bulk PtTe_2_ crystals. The standard XRD pattern of PtTe_2_ (JCPDS # 18-0977) is shown as a reference.

**
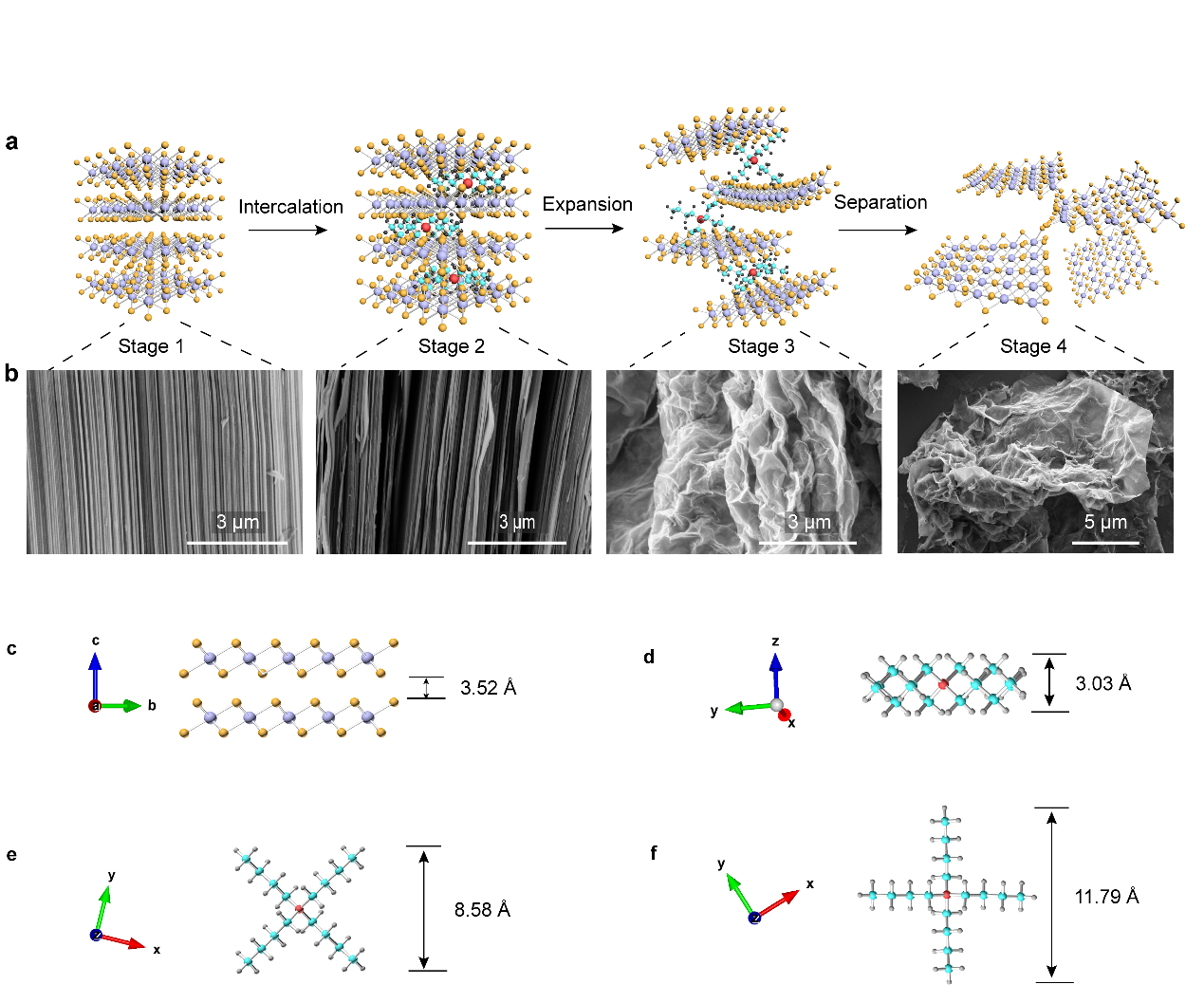
**

**Supplementary Figure 3.** **Electrochemically cathodic exfoliation of bulk PtTe_2_ crystals.** **a**, Schematic illustration showing the mechanism of electrochemical exfoliation of bulk PtTe_2_ crystals in TBAB/DMSO solution. **b**, The corresponding *ex situ* SEM images of pristine bulk PtTe_2_ (stage 1), intercalated PtTe_2_ (stage 2), expanded PtTe_2_ (stage 3), and separated PtTe_2_ (stage 4), respectively.

Electrochemical exfoliation of bulk PtTe_2_ crystals was carried out in a three-electrode system by implementing a constant voltage via chronoamperometry. The morphology of PtTe_2_ crystals during intercalation, expansion, and separation step, were monitored by *ex situ* SEM. The corresponding mechanism was described in Supplementary Fig. 3a. Specifically, when a constant negative bias (-5 V) was applied to the bulk PtTe_2_ crystals (stage 1), TBAB would be intercalated into the interlayer of the bulk PtTe_2_ crystals driven by the electric field (stage 2), which can significantly weaken the van der Waals interaction between the neighboring layers. Afterward, the intercalated TBAB can be electrochemically decomposed into gaseous species^1, 2^, generating the driving force for gigantic and ultrafast volume expansion of the tightly stratified bulk PtTe_2_ crystals (stage 3). Finally, by slight manual shaking, the exfoliated PtTe_2_ could be dispersed into DMSO (stage 4).

**
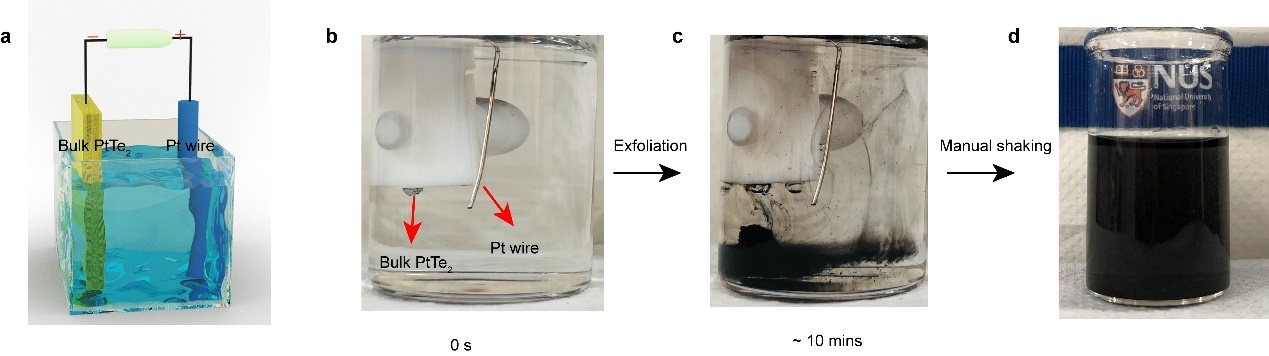
**

**Supplementary Figure 4. Electrochemical exfoliation of bulk PtTe_2_ crystals.** **a**, Schematic illustration showing the electrode system for electrochemical exfoliation of bulk PtTe_2_ crystals in TBAB solution. **b,** Optical photograph of electrochemical exfoliation of bulk PtTe_2_ crystals, where a pristine PtTe_2_ crystal clamped by a Pt electrode. **c,** The obtained PtTe_2_ NSs in DMSO after exfoliation. **d**, The exfoliated PtTe_2_ NSs were dispersed in DMSO via manual shaking.


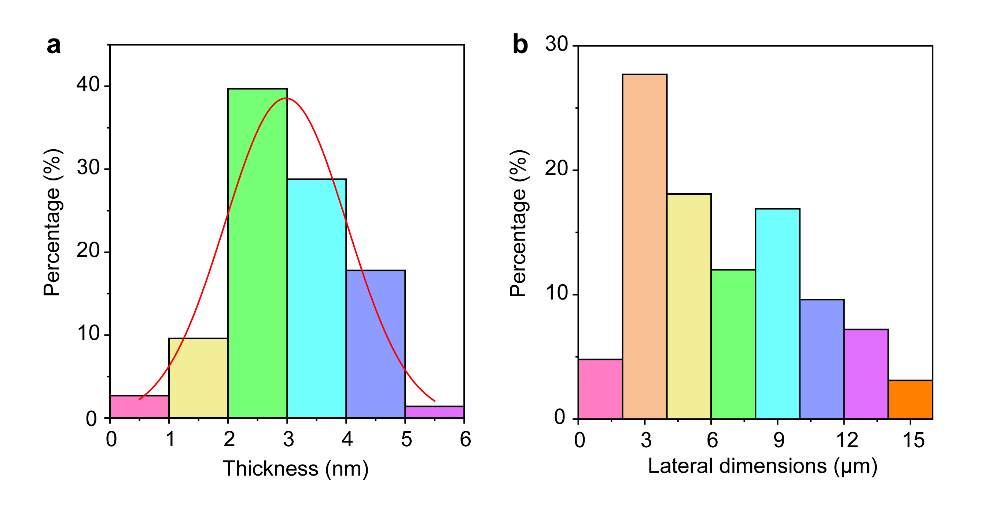


**Supplementary Figure 5. The statistic information on thickness distribution and lateral dimension of exfoliated PtTe_2_ NSs based on AFM results.** The total number of exfoliated PtTe_2_ nanosheets for statistics is about 100. **a**, Thickness distribution of exfoliated PtTe_2_ NSs. **b**, Lateral size distribution of exfoliated PtTe_2_ NSs.

**
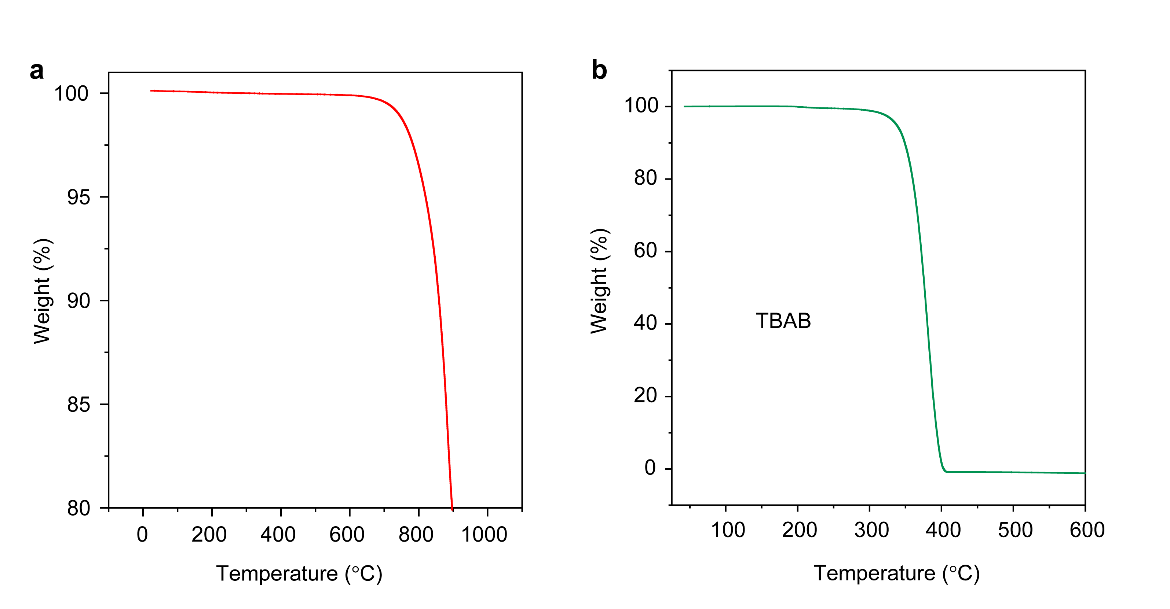
**

**Supplementary Figure 6. TGA results analysis.** **a**, Exfoliated PtTe_2_ NSs. **b**, TBAB molecules.

**
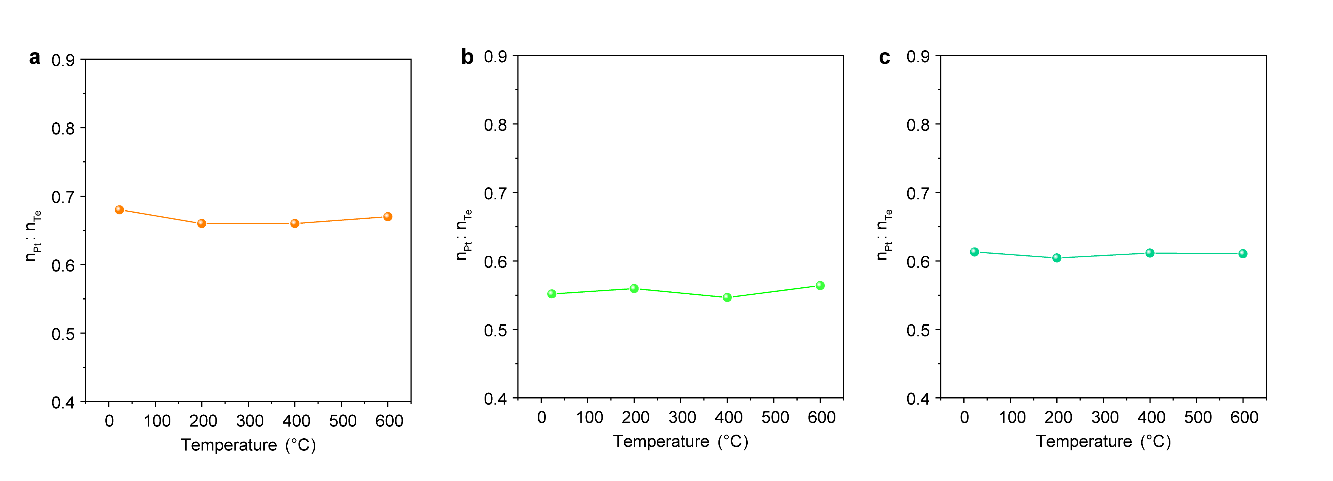
**

**Supplementary Figure 7. The molar ratio of Pt/Te in PtTe_2_.** **a-c**, Three independent n_Pt_/n_Te_ results in PtTe_2_ NSs, PtTe_2_-200 NSs, PtTe_2_-400 NSs, and PtTe_2_-600 NSs.

To rule out the possibility of Te vacancy generation during the thermal treatment process, we repeated the experiments three times from synthesis and electrochemical exfoliation of bulk PtTe_2_ crystals to thermal treatment of PtTe_2_ NSs to obtain PtTe_2_-200 NSs, PtTe_2_-400 NSs, and PtTe_2_-600 NSs. The corresponding samples were examined by ICP-OES. The molar ratio of Pt/Te obtained from the ICP-OES results is shown in Supplementary Figs. 7a-c. It is clear to see that PtTe_2_ NSs keep almost constant atomic ratio of Pt/Te during the heat treatment process. The samples from Experimental 3 (n_Pt_ : n_Te_ = ~0.61, Supplementary Table S1) was used for the measurement of HER performance.

**
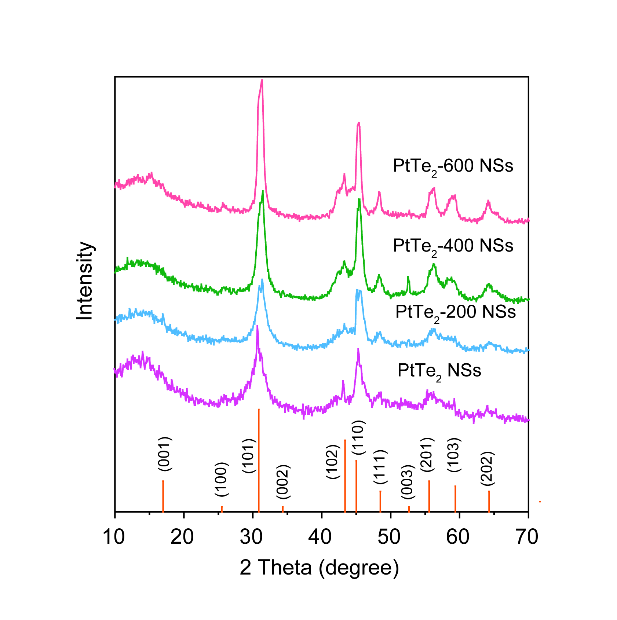
**

**Supplementary Figure 8. XRD results of different PtTe_2_ materials.** XRD patterns of PtTe_2_ NSs, PtTe_2_-200 NSs, PtTe_2_-400 NSs, and PtTe_2_-600 NSs.

**
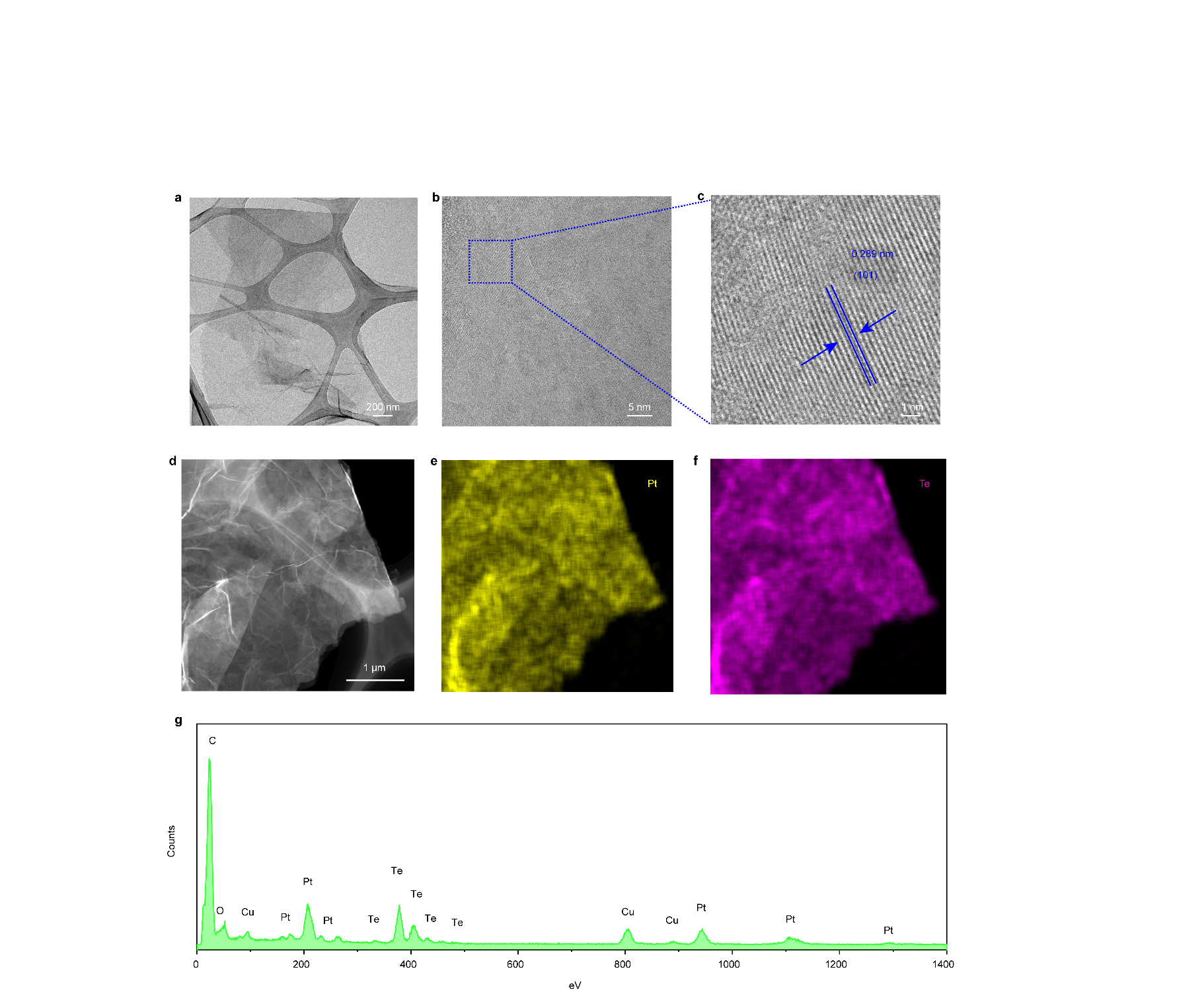
**

**Supplementary Figure 9. TEM characterization of PtTe_2_-200 NSs. a**, TEM image of PtTe_2_-200 NSs. **b**, HRTEM image of PtTe_2_-200 NSs. **c**, Magnified HRTEM image of PtTe_2_-200 NSs. **d-f**, STEM image and the corresponding EDX elemental mapping for Pt and Te in PtTe_2_-200 NSs. **g**, EDX spectrum of PtTe_2_-200 NSs.


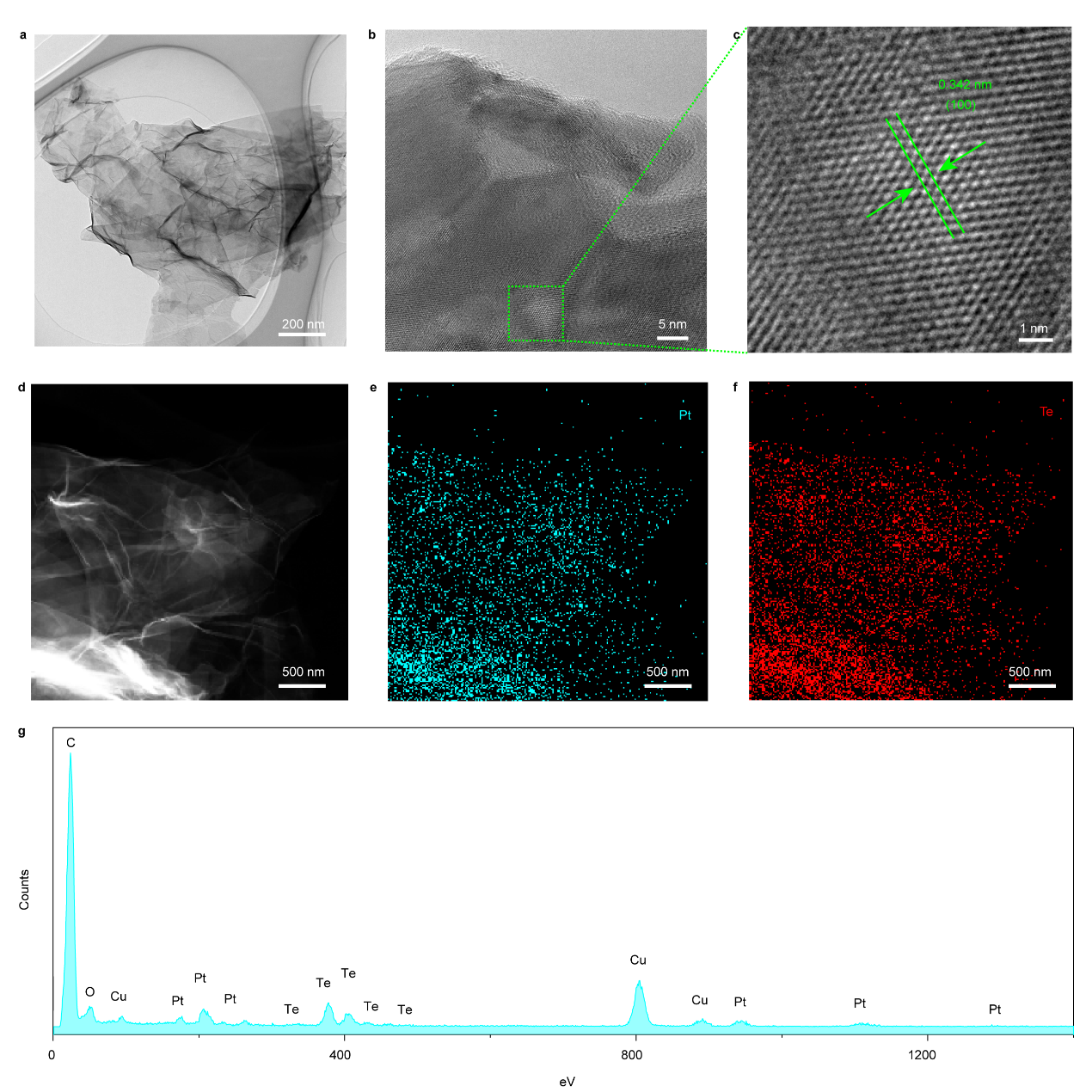


**Supplementary Figure 10. TEM characterization of PtTe_2_-400 NSs. a**, TEM image of PtTe_2_-400 NSs. **b**, HRTEM image of PtTe_2_-400 NSs. **c**, Magnified HRTEM image of PtTe_2_-400 NSs. **d-f**, STEM image and the corresponding EDX elemental mapping for Pt and Te in PtTe_2_-400 NSs. **g**, EDX spectrum of PtTe_2_-400 NSs.

**
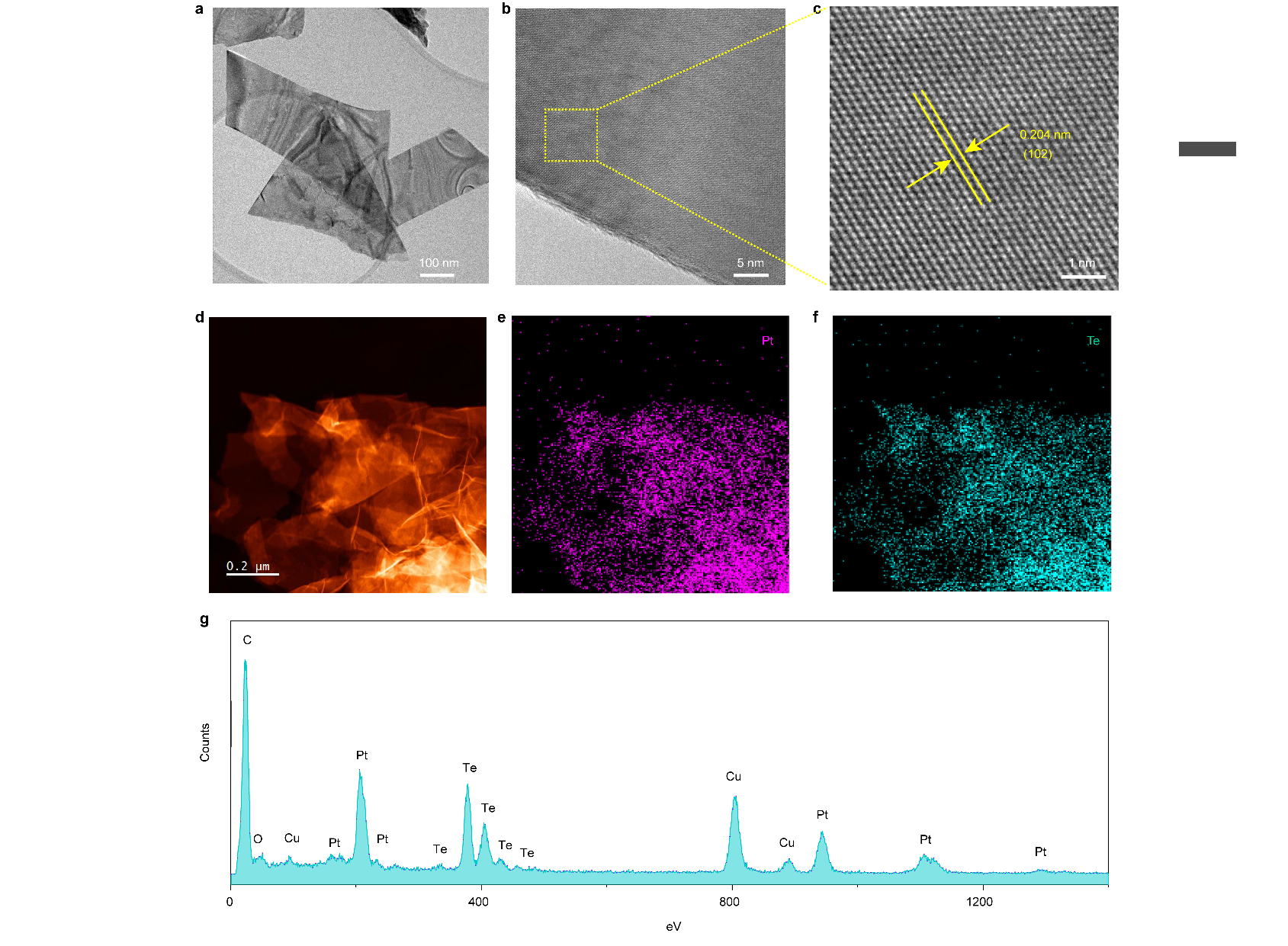
**

**Supplementary Figure 11. TEM characterization of PtTe_2_-600 NSs. a**, TEM image of PtTe_2_-600 NSs. **b**, HRTEM image of PtTe_2_-600 NSs. **c**, Magnified HRTEM image of PtTe_2_-600 NSs. **d-f**, STEM image and the corresponding EDX elemental mapping for Pt and Te in PtTe_2_-600 NSs. **g**, EDX spectrum of PtTe_2_-600 NSs.

**
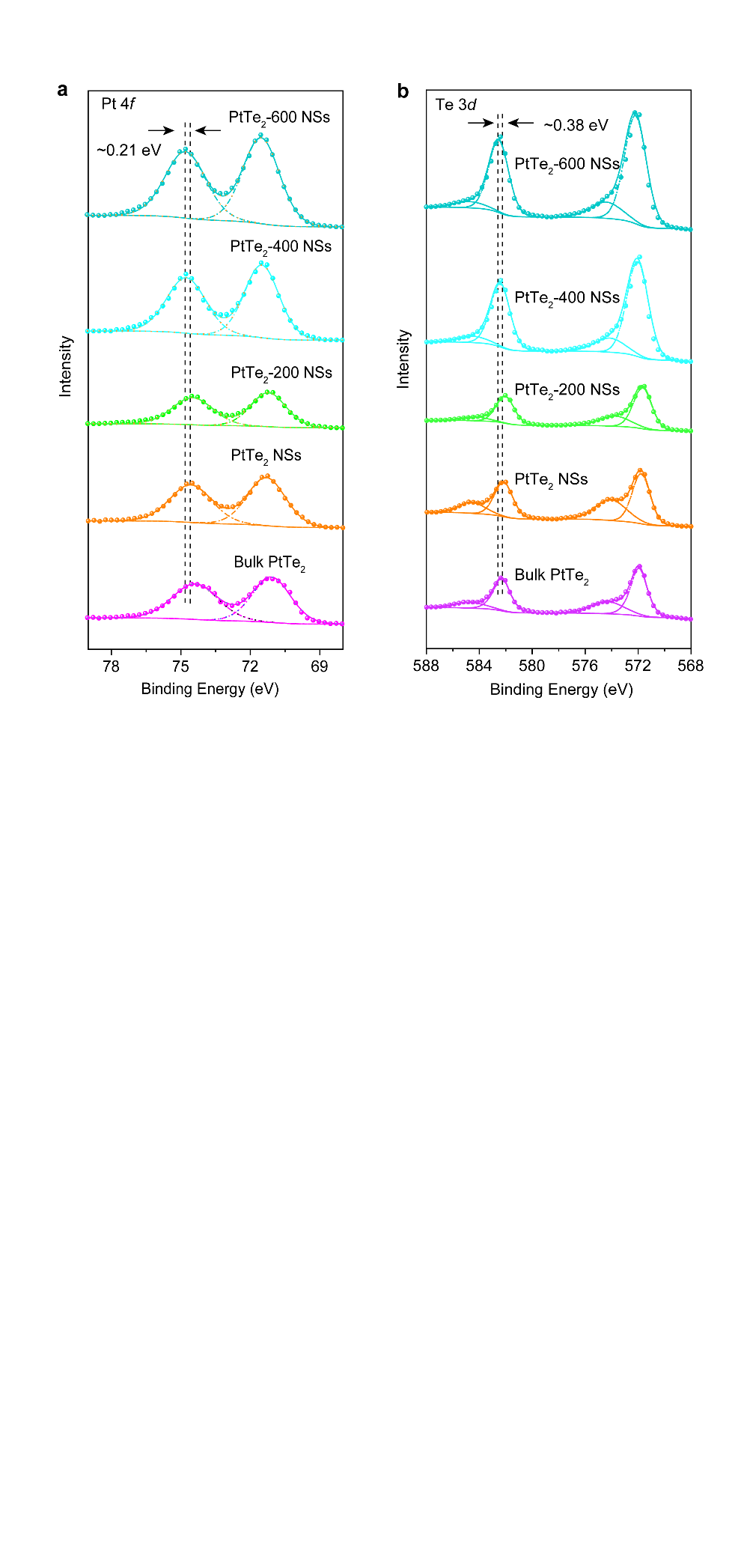
**

**Supplementary Figure 12. XPS spectra of PtTe_2_ samples.** **a**, Pt 4*f* XPS spectra for bulk PtTe_2_, PtTe_2_ NSs, PtTe_2_-200 NSs, PtTe_2_-400 NSs, and PtTe_2_-600 NSs. **b**, Te 3*d* XPS spectra for bulk PtTe_2_, PtTe_2_ NSs, PtTe_2_-200 NSs, PtTe_2_-400 NSs, and PtTe_2_-600 NSs.

**
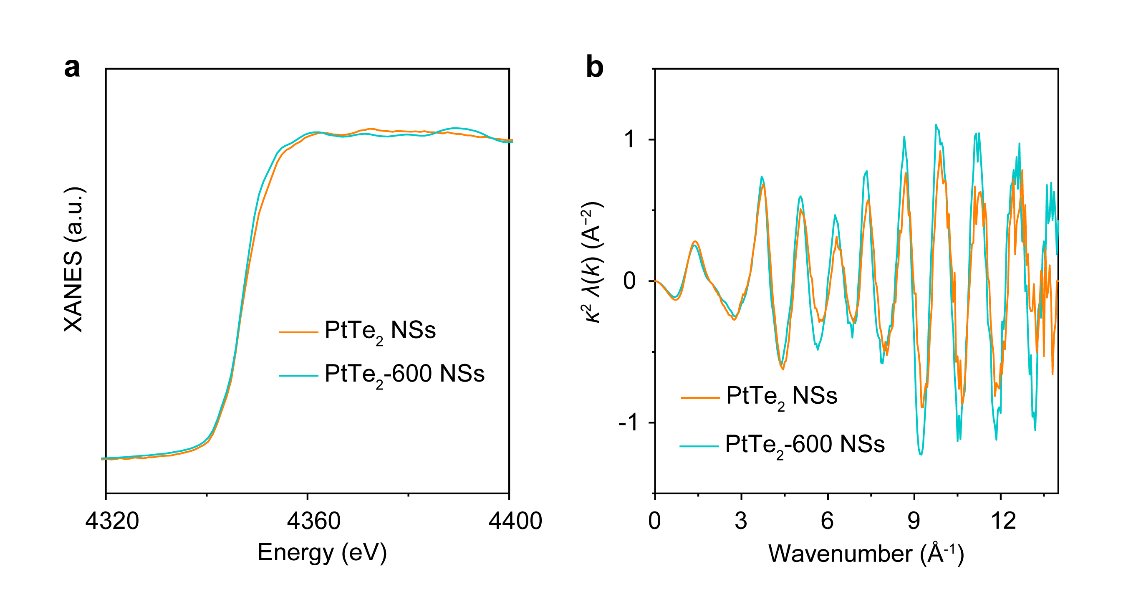
**

**Supplementary Figure 13. XANES and EXAFS results analysis. a**, Normalized Te L_3_-edge XANES spectrum of pristine PtTe_2_ NSs and PtTe_2_-600 NSs. **b**, The corresponding EXAFS *K* space experimental results of PtTe_2_ NSs and PtTe_2_-600 NSs.

**
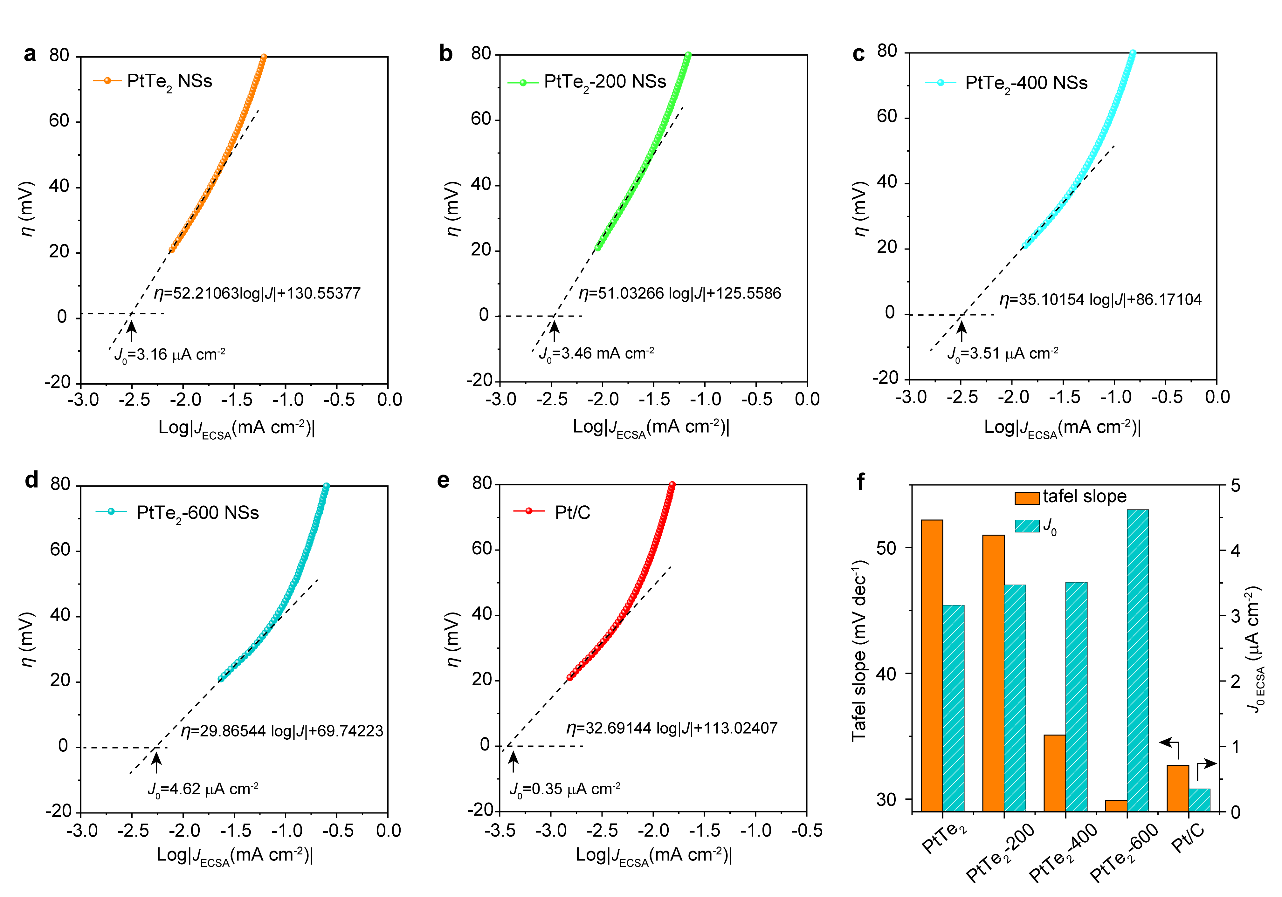
**

**Supplementary Figure 14**. **Tafel plots of different catalysts to determine Tafel slope and exchange current density** **(*J*_0_) based on electrochemically active surface area (ECSA).** **a**, PtTe_2_ NSs. **b**, PtTe_2_-200 NSs. **c**, PtTe_2_-400 NSs. **d**, PtTe_2_-600 NSs. **e**, Pt/C catalysts. **f**, The corresponding histograms of Tafel slope and *J*_0_ based on electrochemically active surface area.

**
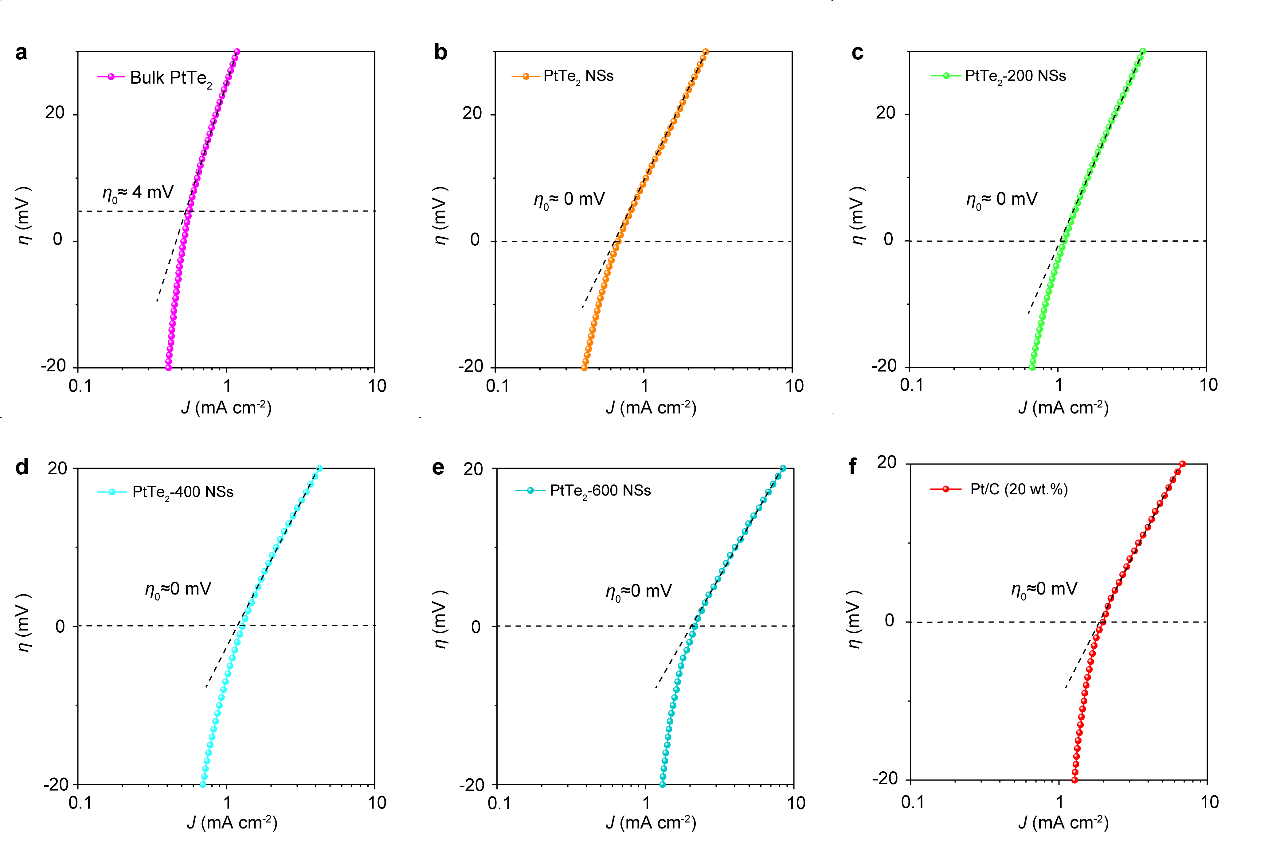
**

**Supplementary Figure 15**. **Onset potential determination of different catalysts.** **a**, bulk PtTe_2_ crystals. **b**, PtTe_2_ NSs. **c**, PtTe_2_-200 NSs. **d**, PtTe_2_-400 NSs. **e**, PtTe_2_-600 NSs. **f**, Pt/C catalyst.

**
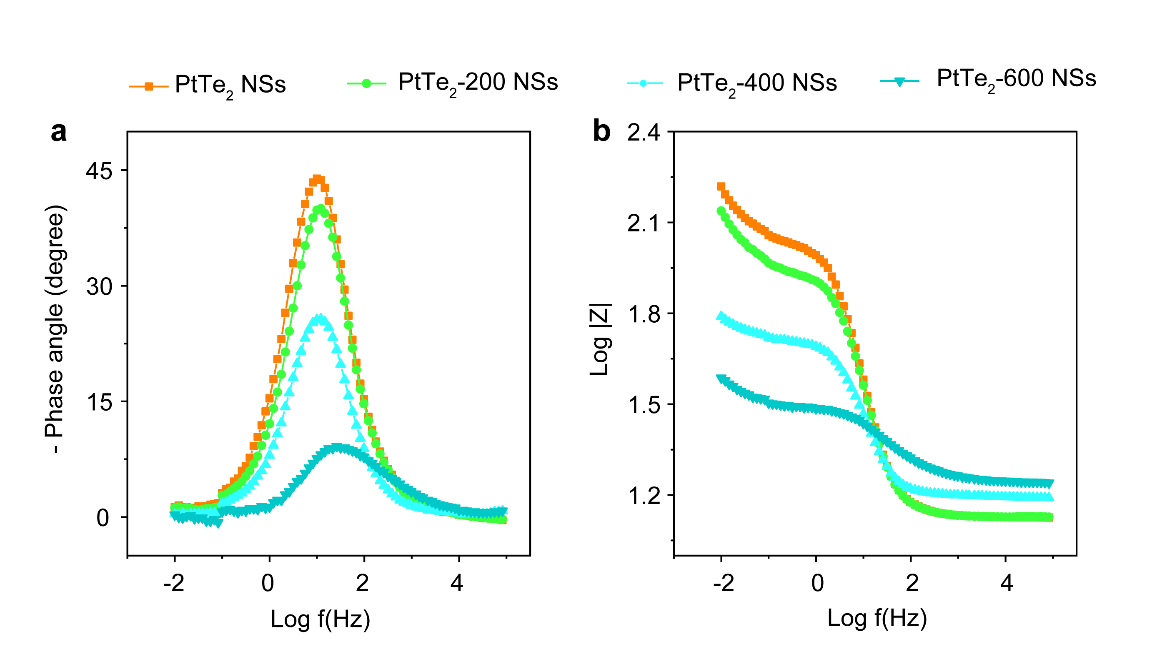
**

**Supplementary Figure 16.** **EIS Bode plots of** **PtTe_2_ NS, PtTe_2_-200 NS, PtTe_2_-400 NS, and PtTe_2_-600 NS. a,** Phase angle plots. **b,** Log|Z| plots.


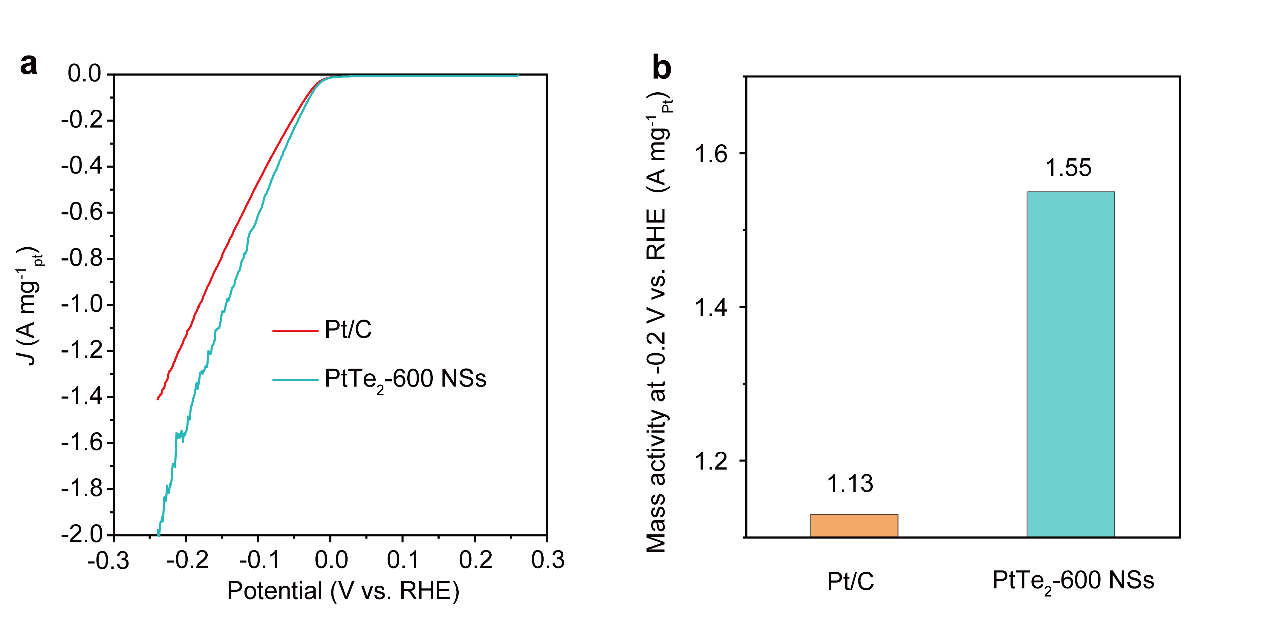


**Supplementary Figure 17. Comparison between PtTe_2_-600 NSs and Pt/C. a** Comparison of LSV curves based on mass activity of Pt in PtTe_2_-600 NSs and Pt/C. **b** Mass activity comparison of Pt between PtTe_2_-600 NSs and Pt/C catalysts at -0.2 V vs. RHE.

**
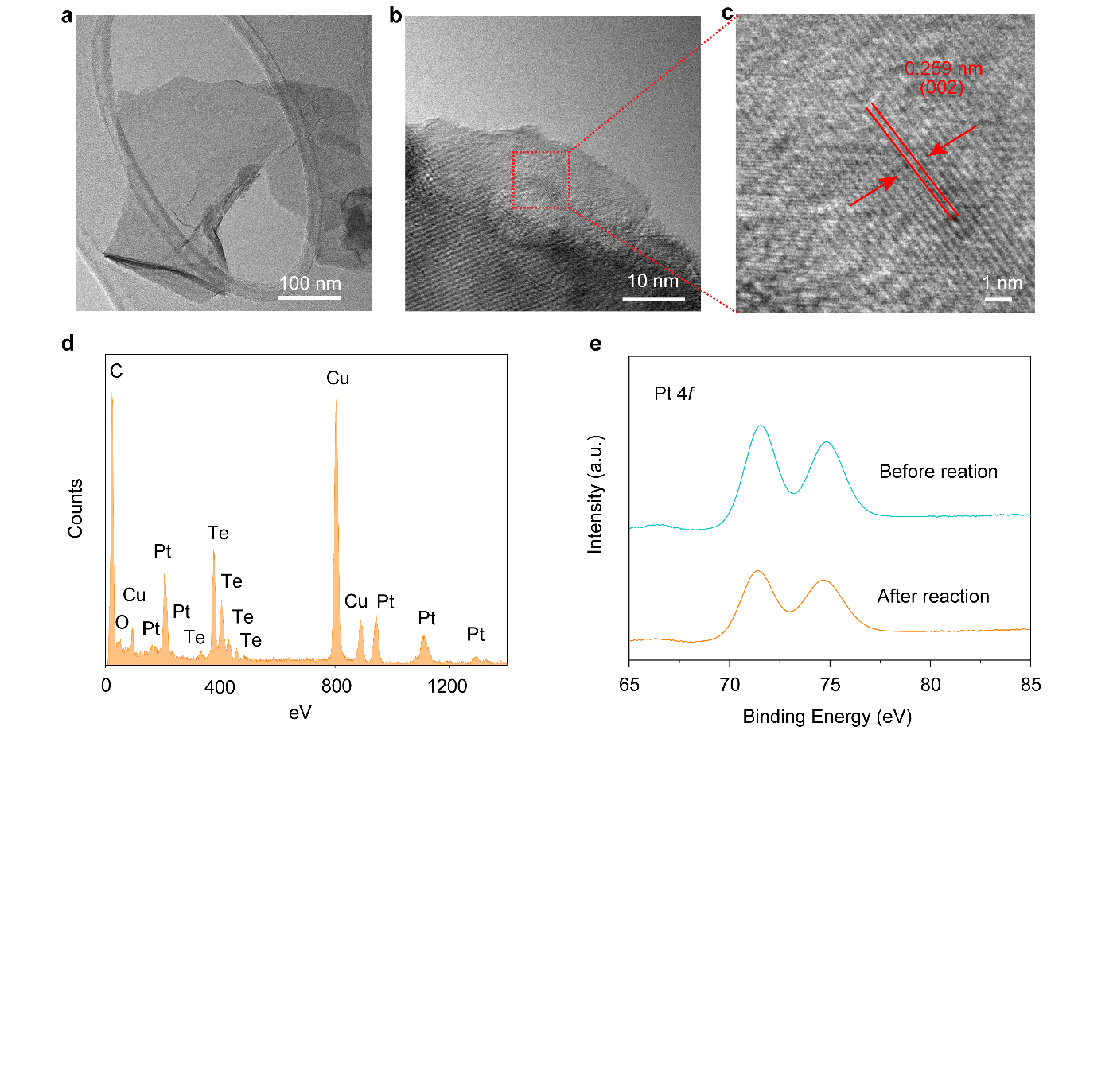
**

**Supplementary Figure 18.** **TEM and XPS characterization of PtTe_2_-600 NSs after stability test.** **a**, TEM image of PtTe_2_-600 NSs after stability test. **b**, HRTEM image of PtTe_2_-600 NSs after stability test. **c**, Magnified HRTEM image of PtTe_2_-600 NSs after stability test. **d**, EDX spectrum of PtTe_2_-600 NSs after stability test. **e**, Pt 4*f* XPS spectra for PtTe_2_-600 NSs after stability test.

**
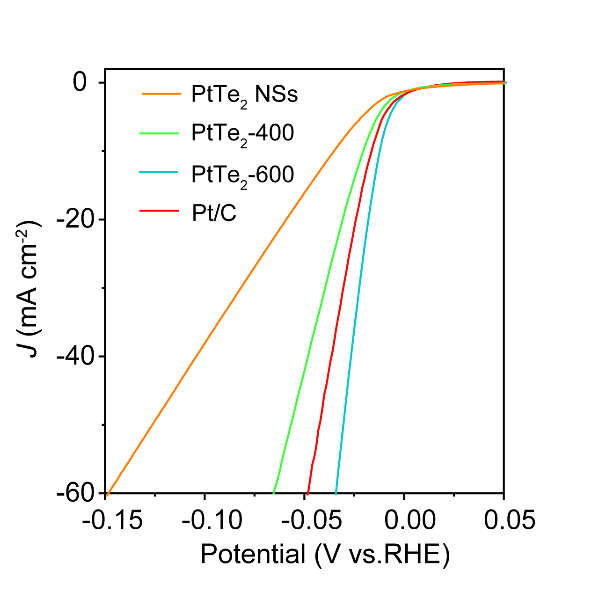
**

**Supplementary Figure 19.** **HER performance in 0.5 M H_2_SO_4_.** LSV curves of PtTe_2_ NSs, PtTe_2_-400 NSs, PtTe_2_-600 NSs, and Pt/C recorded in 0.5 M H_2_SO_4_ electrolyte at a scan rate of 5 mV s^-1^, with 80 % iR compensation.

**
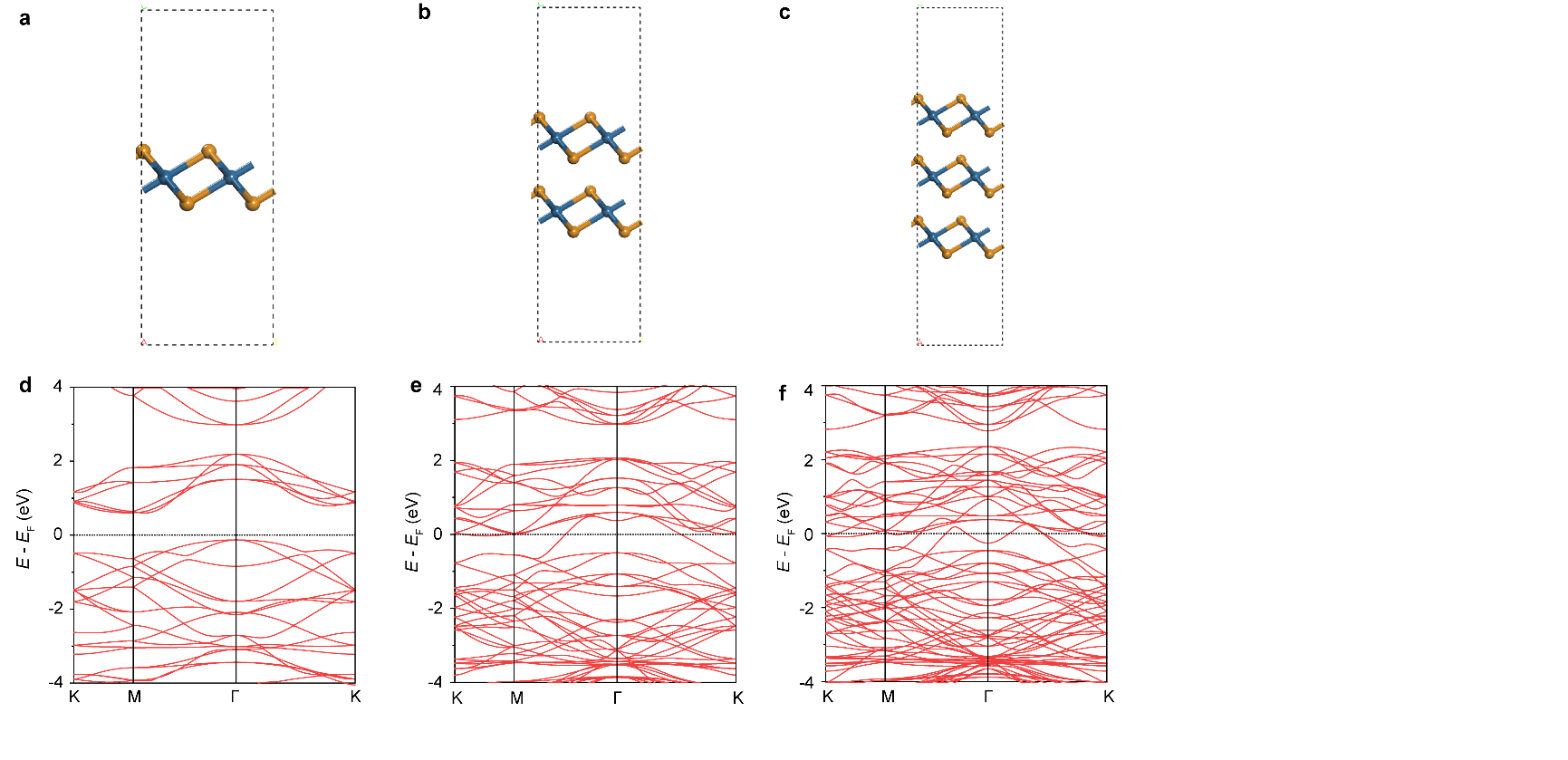
**

**Supplementary Figure 20.** Models and band structure of (**a, d**) monolayer, (**b**, **e**) bilayer and (**c**, **f**) trilayer of 2x2 PtTe_2_ surface. Most of the exfoliated PtTe_2_ NSs have more than 3-layer structures (Supplementary Fig. 5), suggesting that PtTe_2_ NSs have good conductivity. Thus, the conductivity is not the decisive factor for the significantly improved HER performance.

**
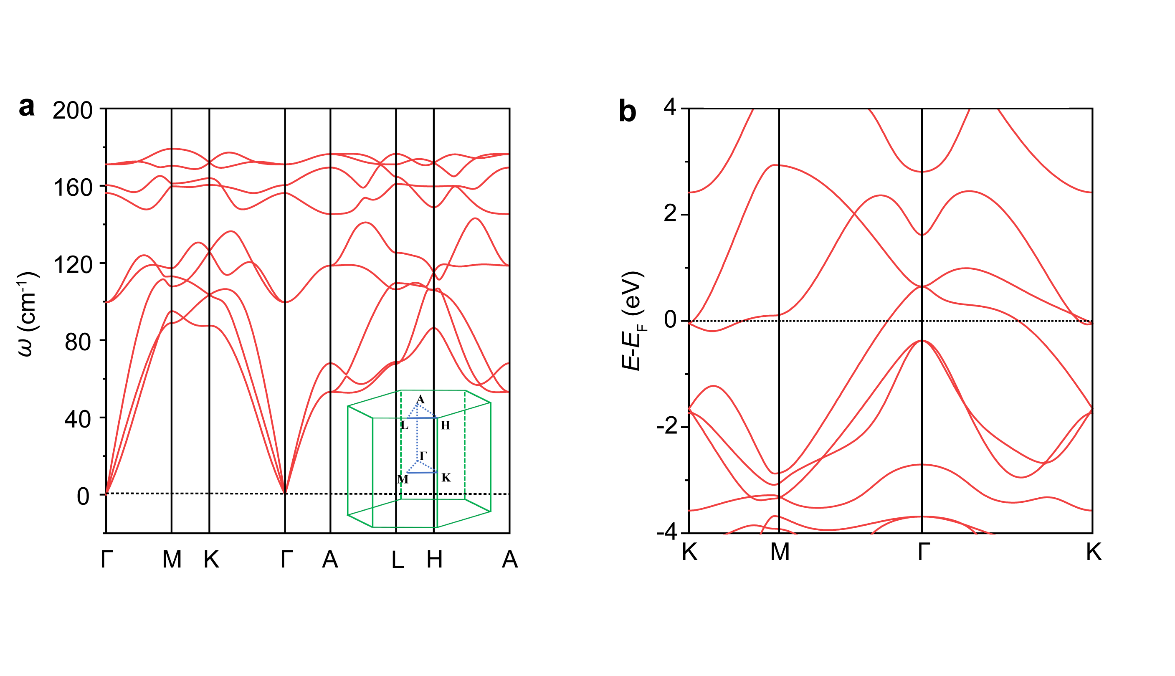
**

**Supplementary Figure 21. Fundamental properties of PtTe_2_ unitcell. a**, Phonon spectra. **b**, Band structure. None of imaginary frequency in **a** indicates the dynamic stability of the optimized PtTe_2_. Fermi energy crossing a band in **b** implies the metal character of bulk PtTe_2_.

**
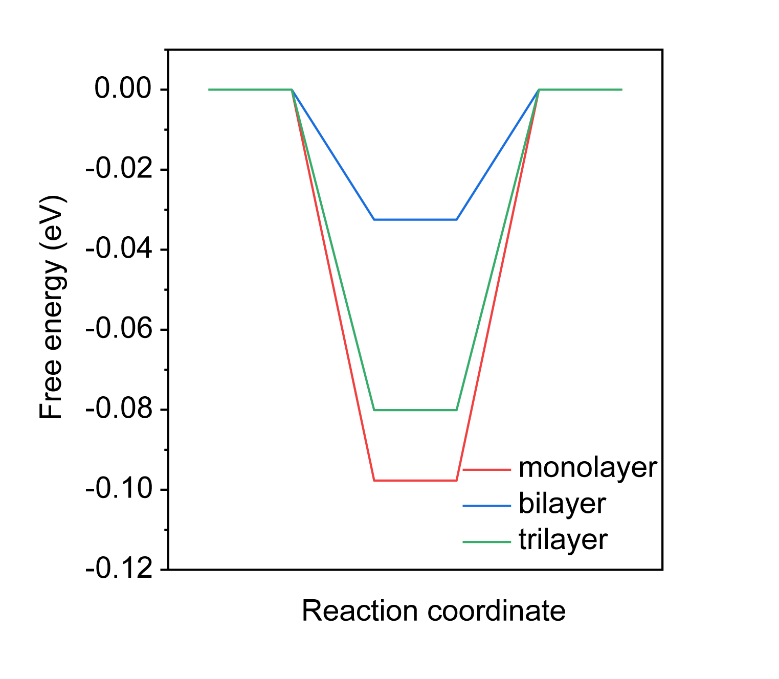
**

**Supplementary Figure 22. Free energy diagram of HER on different layered PtTe_2_.** **a**, Monolayer of 2x2 PtTe_2_ surface. **b**, Bilayer of 2x2 PtTe_2_ surface. **c**, Trilayer of 2x2 PtTe_2_ surface.

**
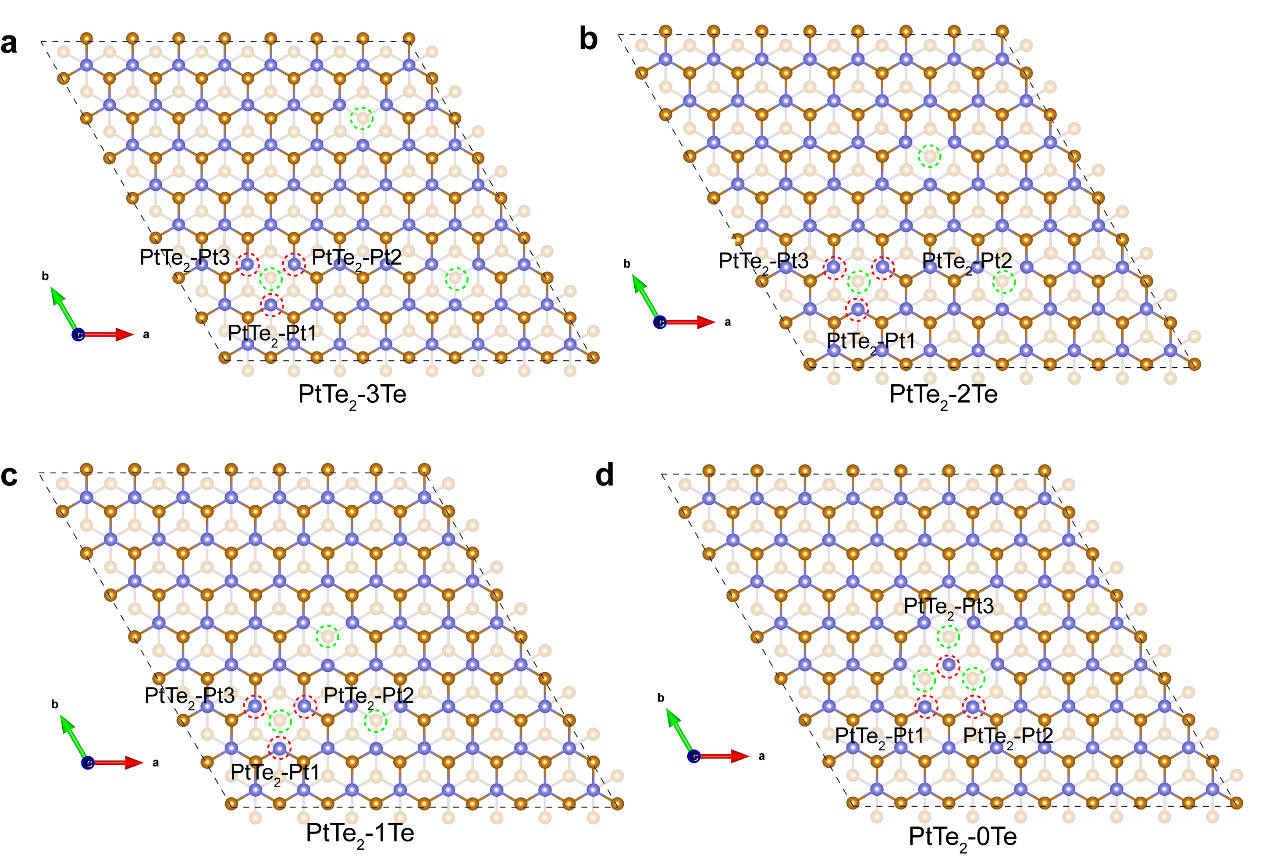
**

**Supplementary Figure 23. Different PtTe_2_ bilayer structures with three Te vacancies, where Te vacancy on the surface is marked as dotted green circles. a,** PtTe_2_-3Te structure, where three Te atoms exist between two Te vacancies. **b**, PtTe_2_-2Te structure, where two Te atoms exist between two Te vacancies. **c**, PtTe_2_-1Te structure, where one Te atom exists between two Te vacancies. **d**, PtTe_2_-0Te structure, where none Te atom exists between two Te vacancies. In addition, different undercoordinated Pt-sites near the Te vacancy are denoted as PtTe_2_-Pty (y = 1, 2, or 3), where “y” is the specific atomic position of Pt atoms, and marked as dotted red circles in the structure model.

**
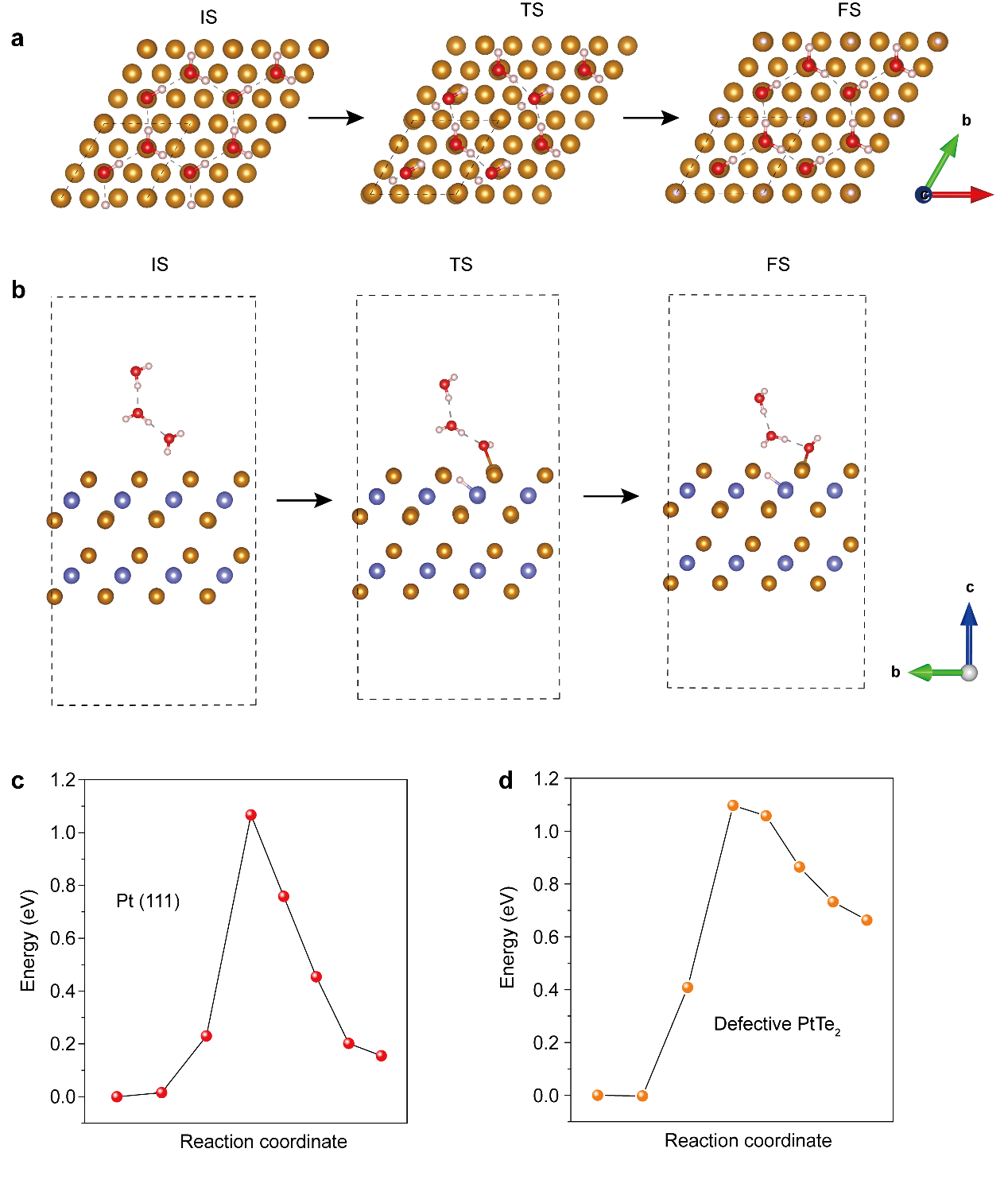
**

**Supplementary Figure 24.** **H_2_O dissociation analysis.** **a,** H_2_O dissociation process on Pt (111), from initial state (IS) to transition state (TS) and then to final state (FS). **b,** H_2_O dissociation on defective PtTe_2_, from IS to TS and then to FS. **c,** The corresponding energy barrier of H_2_O dissociation on Pt (111) (1.07 eV). **d,** The corresponding energy barrier of H_2_O dissociation on defective PtTe_2_ (1.09 eV). For the defective PtTe_2_, three H_2_O molecular was adopted to model the water environment, while for the Pt (111) surface, a commonly used bi-layer H_2_O adsorption structure was adopted^3-5^.

**
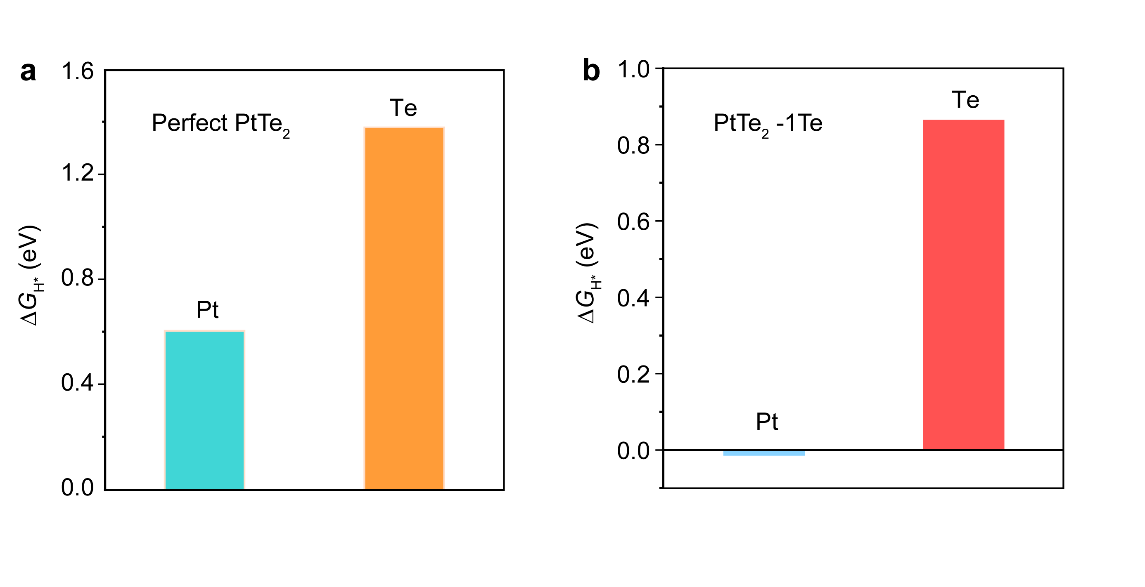
**

**Supplementary Figure 25. Δ*G*_H*_ analysis of different PtTe_2_.** **a**, Δ*G*_H*_ of Pt sites and Te sites in PtTe_2_ without defects. **b**, Δ*G*_H*_ of Pt sites and Te sites in PtTe_2_-1Te structure.

**
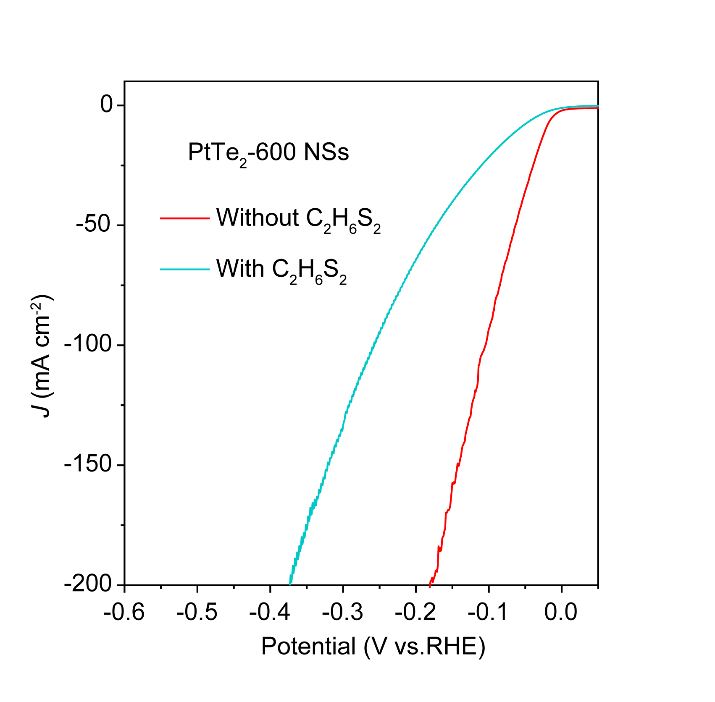
**

**Supplementary Figure 26.** HER performances of PtTe_2_-600 NSs with and without the addition of C_2_H_6_S_2_ in 1.0 M KOH.

The significantly decreased HER activity of PtTe_2_-600 NSs after addition of C_2_H_6_S_2_ reveals that C_2_H_6_S_2_ molecular can effectively poison Pt sites in PtTe_2_-600 NSs due to the strong coordination ability between Pt and S atoms. The results reveal that the Pt atoms in PtTe_2_-600 NSs are the active sites for HER, rather than the Te atoms.

**
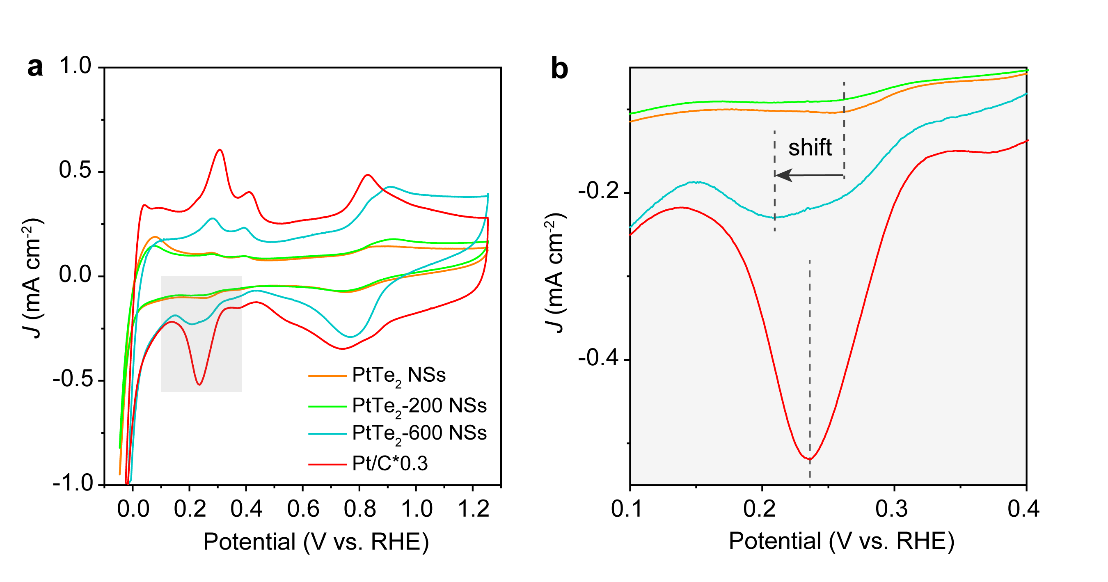
**

**Supplementary Figure 27. Underpotentially deposited hydrogen (H_upd_) peak analysis.** **a**, CV curves of PtTe_2_ NS, PtTe_2_-200 NS, PtTe_2_-600 NS and Pt/C measured in Argon purged 1.0 M KOH at a scan rate of 50 mV s^-1^. **b**, Enlarged CV curves of gray area in **a**.


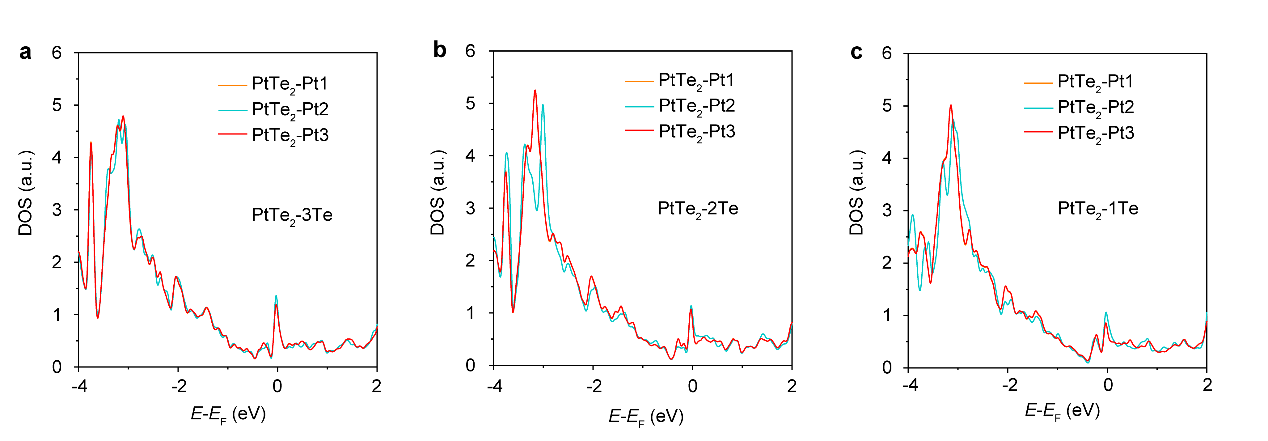


**Supplementary Figure 28.** **DOS analysis.** **a**, PtTe_2_-3Te structure. **b**, PtTe_2_-2Te structure. **c**, PtTe_2_-1Te structure. The orange, blue, and red lines represent the DOS of Pt1, Pt2, and Pt3 sites in the corresponding PtTe_2_-XTe structure, respectively.

**
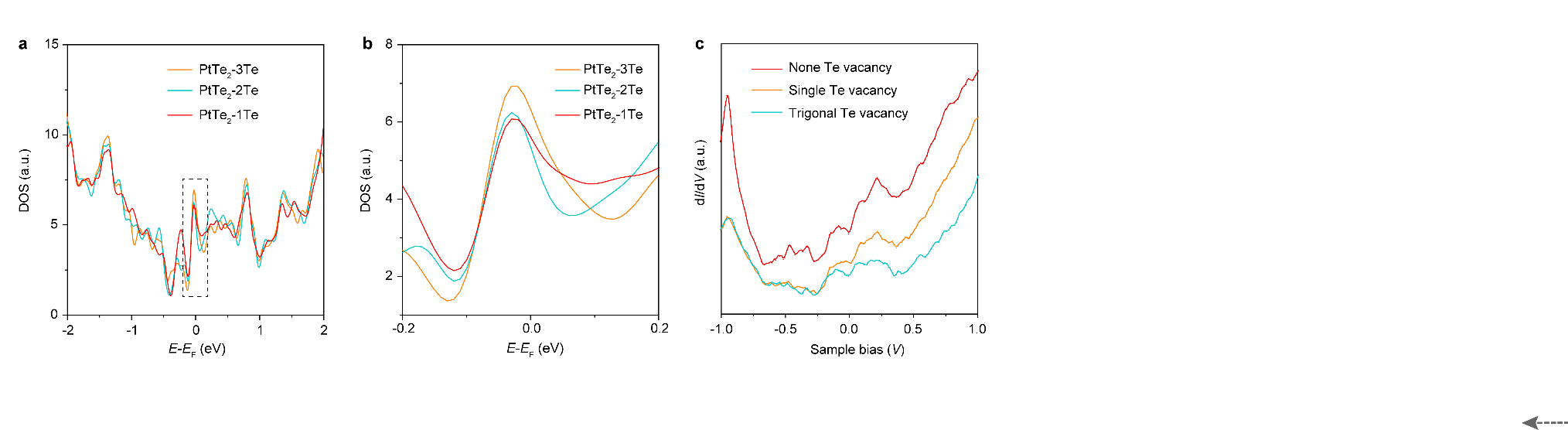
**

**Supplementary Figure 29.** **DOS calculations and STS experimental results.** **a**, Total DOS of Pt and Te atoms around the defect in PtTe_2_-3Te, PtTe_2_-2Te, and PtTe_2_-1Te structure. **b**, Magnified DOS profile in the dotted box in **a**, showing gradually decreased total DOS from PtTe_2_-3Te, PtTe_2_-2Te, to PtTe_2_-1Te structure. **c**, d*I*/d*V* spectrum at near-Fermi region to show local DOS of none-vacancy terrace, single-Te vacancy and trigonal-Te vacancy in PtTe_2_ (marked to red, orange, and blue symbols, respectively, in Fig. 2d).


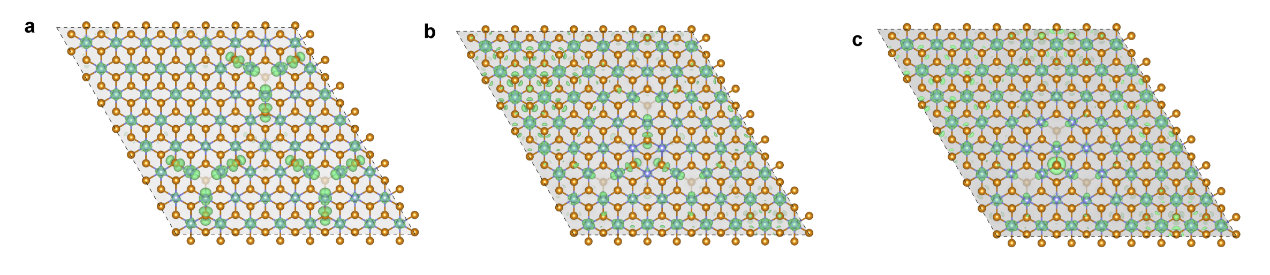


**Supplementary Figure 30.** **Partial charge of different defective PtTe_2_ around Fermi level**. **a**, PtTe_2_-3Te structure, **b**, PtTe_2_-2Te structure and **c**, PtTe_2_-1Te structure.

**
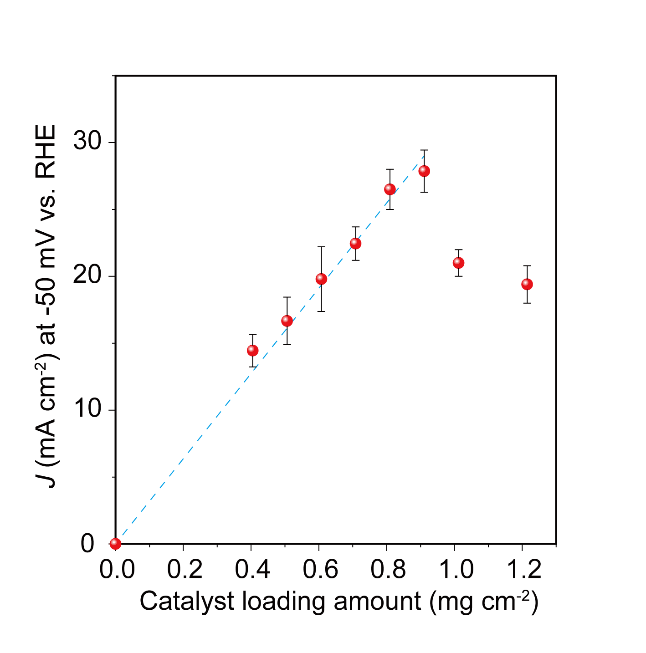
**

**Supplementary Figure 31. Catalyst loading amount dependent current density analysis.** Plot of Pt/C (20 wt.%) catalyst loading amount vs. current density at a fixed overpotential of 50 mV. Error bar represents the mean deviation.

**
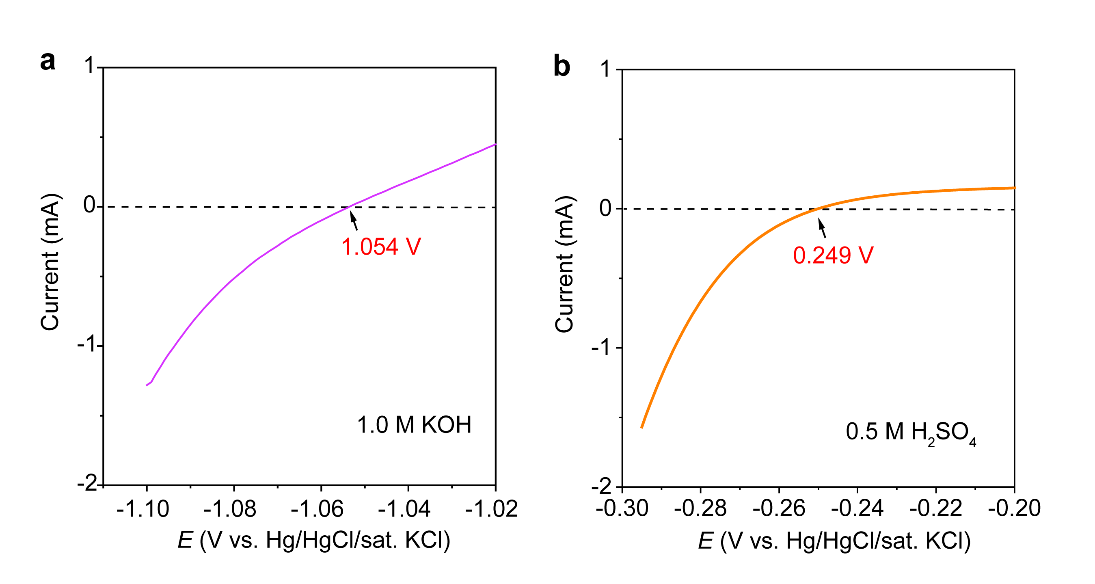
**

**Supplementary Figure 32.** **Reference electrode calibration based on the reported method**^6-9^**. a,** LSV curve of Pt in 1.0 M KOH solution (H_2_-saturated), used for calibration of saturated calomel electrode with respect to RHE. **b,** LSV curve of Pt in 0.5 M H_2_SO_4_ solution (H_2_-saturated), used for calibration of saturated calomel electrode with respect to RHE. Scan rate: 5 mV s^-1^. Two Pt sheets and a Hg/HgCl electrode were used as the counter, working, and reference electrode, respectively. In 1.0 M KOH, *E*(RHE) = *E*(vs. Hg/HgCl) + 1.054 V; In 0.5 M H_2_SO_4_, *E*(RHE) = *E*(vs. Hg/HgCl) + 0.249 V.

**Supplementary Table S1.** ICP–OES results showing the molar ratio of Pt/Te in PtTe_2_.

| Sample | n(Pt) : n(Te) | | | |
| --- | --- | --- | --- | --- |
|  | PtTe_2_ NSs | PtTe_2_-200 NSs | PtTe_2_-400 NSs | PtTe_2_-600 NSs |
| Experiment 1 | 0.68 | 0.66 | 0.66 | 0.67 |
| Experiment 2 | 0.55 | 0.56 | 0.55 | 0.56 |
| Experiment 3 | 0.61 | 0.60 | 0.61 | 0.61 |

Notes: the molar ratio of Pt : Te in the prepared PtTe_2_ catalysts is larger than the theoretical stoichiometric ratio of PtTe_2_ (0.5) according to the ICP-OES results, which means that there exist Te vacancies in the prepared PtTe_2_ catalysts. When n_Pt_ : n_Te_ in PtTe_2_ nanosheets is about 0.61, the atomic percentage of Te vacancies is calculated to be 18 at.%.

**Supplementary Table S2.** Comparison of HER performance in 1.0 M KOH

| Catalyst | Loading amount (μg cm^-2^) | η (mV)@10 mA cm^-2^ | Tafel slope | Ref. |
| --- | --- | --- | --- | --- |
| PtTe_2_-600 NSs | 336 | 22 | 29.9 | This work |
| Pt/C (20%) | 815 | 26 | 32.7 | This work |
| Pt@MXene | 200 | 27 | 41 | ^10^ |
| PtNi-O/C ^a^ | 5.1 | 39.8 | 78.8 | ^11^ |
| Pt_1_/N-C | 250 | 46 | 39.8 | ^12^ |
| Pt-Ni ASs ^a^ | NA | 27.7 | 27 | ^13^ |
| Pt/np-Co_0.85_Se ^a^ | 2040 | 58 | 39 | ^14^ |
| Pt-NC/Ni-MOF | NA | 25 | 42.1 | ^15^ |
| Pt-NG/C ^a^ | 283 | 35.28 | 27 | ^16^ |
| PtRu NCs/BP ^a^ | NA | 64 | 43 | ^17^ |
| Pt@PCM ^a^ | NA | 139 | 73.6 | ^18^ |
| er-WS_2_-Pt ^a^ | 640 | 48 | 65 | ^19^ |
| Pt+Ni(HCO_3_)_2_ ^a^ | NA | 27 | 45 | ^20^ |
| PtSn_4_ single crystal ^a^ | NA | 37 | 39 | ^21^ |
| Pt_3_Ni_2_ NWs-S/C ^a^ | 15 | 42 | NA | ^22^ |
| hcp Pt–Ni alloy ^a^ | NA | 65 | 78 | ^23^ |
| Pt_5_/HMCS | 7.6 | 46.2 | 48.1 | ^24^ |
| Bped-Pt/GR ^a^ | NA | 21 | 46.9 | ^25^ |
| Ir@CON | ~ 500 | 19.2 | 29 | ^26^ |
| Ir_1_@Co/NC | NA | 55 | 119 | ^27^ |
| Pt_3_Ni_3_ NWs/C-air ^a^ | NA | 40 | NA | ^28^ |
| IrCo@NC ^a^ | 285 | 45 | 80 | ^29^ |
| RhPd-H/C ^a^ | NA | 40 | 35.7 | ^30^ |
| RuP_2_ | 1000 | 52 | 59 | ^31^ |
| RuCo nanoalloys ^a^ | 275 | 28 | 31 | ^32^ |
| Ru@MWCNT | 160 | 13 | 27 | ^33^ |
| Ru-NC-700 | 200 | 12 | NA | ^34^ |
| Co-substituted Ru | 153 | 13 | 29 | ^8^ |
| Ru_2_P/RGO ^a^ | 1000 | 13 | 56 | ^35^ |
| Ru/C-300 ^a^ | NA | 14 | 32.5 | ^36^ |
| Ru/CN-800 ^a^ | 498 | 14 | 30 | ^37^ |
| Ru/3DNPC-500 ^a^ | NA | 15 | 31 | ^38^ |
| Ru@C_2_N | ~285 | 17 | 38 | ^7^ |
| RuP (L-RP)^a^ | 464 | 18 | 34 | ^39^ |
| Ru/NC | NA | 21 | 31 | ^40^ |
| Ru@GnP | 250 | 22 | 28 | ^41^ |
| 4H/fcc Ru NTs | 124 | 23 | 29.4 | ^42^ |
| Ru/C ^a^ | ~590 | 24 | 33 | ^43^ |
| s-RuS_2_/S-rGO ^a^ | 464 | 25 | 29 | ^44^ |
| Cu-doped Ru-RuO_2_/C ^a^ | 285 | 28 | 35 | ^45^ |
| Ru-Ni@Ni_2_P-HNRs ^a^ | NA | 31 | 41 | ^46^ |
| Ru@CN ^a^ | NA | 32 | 53 | ^47^ |
| Ru-NGC ^a^ | 360 | 37 | 40 | ^48^ |
| CNx@Ru/MWCNT ^a^ | NA | 39 | 28 | ^49^ |
| RuO_2_/N−C ^a^ | NA | 40 | 44 | ^50^ |
| Ru ND/C | NA | 43.4 | 49 | ^51^ |
| CoRu@NC ^a^ | 273 | 45 | 66 | ^52^ |
| Sr_2_RuO_4_ | 464 | 61 | 51 | ^53^ |
| RuPx@NPC ^a^ | NA | 74 | 70 | ^54^ |
| RuO_2_-NWs@g-CN ^a^ | NA | 95 | 70 | ^55^ |
| Au-Ru-2 NWs | 80 | 25 | 15.6 | ^56^ |
| a-RuTe_2_ PNRs ^a^ | NA | 36 | 36 | ^57^ |
| RhPd-H NPs | NA | 36.6 | 35.3 | ^58^ |
| Ni-Co/1T phase MoS_2_ | 183 | 70 | 38 | ^59^ |
| SV-MoS_2_ ^a^ | NA | 170 | 60 | ^60^ |
| M-MoS_2_ | 43 | 175 | 41 | ^61^ |
| C-MoS_2_ ^a^ | NA | 45 | 46 | ^62^ |
| MoP@NCHSs-900 ^a^ | 400 | 92 | 62 | ^63^ |
| NFP/C-3 | 400 | 95 | 72 | ^64^ |
| Mo_2_N-Mo_2_C | 337 | 154 | 68 | ^65^ |
| CoN_x_/C | 2000 | 170 | 75 | ^66^ |
| MoC_x_ ^a^ | 800 | 151 | 59 | ^67^ |

**^a^** Information about reference electrode calibration is not mentioned in the journal.

**References**

1. Li J*, et al.* Ultrafast electrochemical expansion of black phosphorus toward high-yield synthesis of few-layer phosphorene. *Chem. Mater.* **30**, 2742-2749 (2018).

2. Fang Y*, et al.* Janus electrochemical exfoliation of two-dimensional materials. *J. Mater. Chem. A* **7**, 25691-25711 (2019).

3. Zheng Y*, et al.* High electrocatalytic hydrogen evolution activity of an anomalous ruthenium catalyst. *J. Am. Chem. Soc.* **138**, 16174-16181 (2016).

4. Hossain MD, Huang Y, Yu TH, Goddard Iii WA, Luo Z. Reaction mechanism and kinetics for CO_2_ reduction on nickel single atom catalysts from quantum mechanics. *Nat. Commun.* **11**, 2256 (2020).

5. Feibelman PJ. Partial dissociation of water on Ru(0001). *Science* **295**, 99 (2002).

6. Chen C-H*, et al.* Ruthenium-based single-atom alloy with high electrocatalytic activity for hydrogen evolution. *Adv. Energy Mater.* **9**, 1803913 (2019).

7. Mahmood J*, et al.* An efficient and pH-universal ruthenium-based catalyst for the hydrogen evolution reaction. *Nat. Nanotechnol.* **12**, 441-446 (2017).

8. Mao J*, et al.* Accelerating water dissociation kinetics by isolating cobalt atoms into ruthenium lattice. *Nat. Commun.* **9**, 4958 (2018).

9. Xie Y*, et al.* Boosting water dissociation kinetics on Pt–Ni nanowires by n-induced orbital tuning. *Adv. Mater.* **31**, 1807780 (2019).

10. Xiu L*, et al.* Multilevel hollow Mxene tailored low-Pt catalyst for efficient hydrogen evolution in full-pH range and seawater. *Adv. Funct. Mater.*, 1910028 (2020).

11. Zhao Z*, et al.* Surface-engineered PtNi-O nanostructure with record-high performance for electrocatalytic hydrogen evolution reaction. *J. Am. Chem. Soc.* **140**, 9046-9050 (2018).

12. Fang S*, et al.* Uncovering near-free platinum single-atom dynamics during electrochemical hydrogen evolution reaction. *Nat. Commun.* **11**, 1029 (2020).

13. Zhang Z*, et al.* Crystal phase and architecture engineering of lotus-thalamus-shaped Pt-Ni anisotropic superstructures for highly efficient electrochemical hydrogen evolution. *Adv. Mater.* **30**, 1801741 (2018).

14. Jiang K*, et al.* Single platinum atoms embedded in nanoporous cobalt selenide as electrocatalyst for accelerating hydrogen evolution reaction. *Nat. Commun.* **10**, 1743 (2019).

15. Guo C, Jiao Y, Zheng Y, Luo J, Davey K, Qiao S-Z. Intermediate modulation on noble metal hybridized to 2d metal-organic framework for accelerated water electrocatalysis. *Chem* **5**, 2429-2441 (2019).

16. Sun M*, et al.* Overwhelming the performance of single atoms with atomic clusters for platinum-catalyzed hydrogen evolution. *ACS Catal.* **9**, 8213-8223 (2019).

17. Li Y*, et al.* Hybrids of PtRu nanoclusters and black phosphorus nanosheets forhighly efficient alkaline hydrogen evolution reaction. *ACS Catal.* **9**, 10870-10875 (2019).

18. Zhang H*, et al.* Dynamic traction of lattice-confined platinum atoms into mesoporous carbon matrix for hydrogen evolution reaction. *Sci. Adv.* **4**, eaao6657 (2018).

19. Tang K, Wang X, Li Q, Yan C. High edge selectivity of in situ electrochemical Pt deposition on edge-rich layered WS_2_ nanosheets. *Adv. Mater.* **30**, 1704779 (2018).

20. Lao M*, et al.* Platinum/nickel bicarbonate heterostructures towards accelerated hydrogen evolution under alkaline conditions. *Angew. Chem. Int. Ed.* **58**, 5432-5437 (2019).

21. Li GW*, et al.* Dirac nodal arc semimetal PtSn_4_: An ideal platform for understanding surface properties and catalysis for hydrogen evolution. *Angew. Chem. Int. Edit.* **58**, 13107-13112 (2019).

22. Wang P*, et al.* Precise tuning in platinum-nickel/nickel sulfide interface nanowires for synergistic hydrogen evolution catalysis. *Nat. Commun.* **8**, 14580 (2017).

23. Cao Z*, et al.* Platinum-nickel alloy excavated nano-multipods with hexagonal close-packed structure and superior activity towards hydrogen evolution reaction. *Nat. Commun.* **8**, 15131 (2017).

24. Wan X-K, Wu HB, Guan BY, Luan D, Lou XW. Confining sub-nanometer Pt clusters in hollow mesoporous carbon spheres for boosting hydrogen evolution activity. *Adv. Mater.* **32**, 1901349 (2020).

25. Wang X*, et al.* Rapid activation of platinum with black phosphorus for efficient hydrogen evolution. *Angew. Chem. Int. Ed.* **58**, 19060-19066 (2019).

26. Mahmood J*, et al.* Encapsulating iridium nanoparticles inside a 3d cage-like organic network as an efficient and durable catalyst for the hydrogen evolution reaction. *Adv. Mater.* **30**, 1805606 (2018).

27. Lai W-H*, et al.* General π-electron-assisted strategy for Ir, Pt, Ru, Pd, Fe, Ni single-atom electrocatalysts with bifunctional active sites for highly efficient water splitting. *Angew. Chem. Int. Ed.* **58**, 11868-11873 (2019).

28. Wang PT, Jiang KZ, Wang GM, Yao JL, Huang XQ. Phase and interface engineering of platinum-nickel nanowires for efficient electrochemical hydrogen evolution. *Angew. Chem. Int. Edit.* **55**, 12859-12863 (2016).

29. Jiang P*, et al.* Tuning the activity of carbon for electrocatalytic hydrogen evolution via an iridium-cobalt alloy core encapsulated in nitrogen-doped carbon cages. *Adv. Mater.* **30**, 1705324 (2018).

30. Fan J*, et al.* Hydrogen stabilized RhPdH 2D bimetallene nanosheets for efficient alkaline hydrogen evolution. *J. Am. Chem. Soc.* **142**, 3645-3651 (2020).

31. Pu Z, Amiinu IS, Kou Z, Li W, Mu S. RuP_2_-based catalysts with platinum-like activity and higher durability for the hydrogen evolution reaction at all ph values. *Angew. Chem. Int. Ed.* **56**, 11559-11564 (2017).

32. Su J, Yang Y, Xia G, Chen J, Jiang P, Chen Q. Ruthenium-cobalt nanoalloys encapsulated in nitrogen-doped graphene as active electrocatalysts for producing hydrogen in alkaline media. *Nat. Commun.* **8**, 14969 (2017).

33. Kweon DH*, et al.* Ruthenium anchored on carbon nanotube electrocatalyst for hydrogen production with enhanced Faradaic efficiency. *Nat. Commun.* **11**, 1278 (2020).

34. Lu B*, et al.* Ruthenium atomically dispersed in carbon outperforms platinum toward hydrogen evolution in alkaline media. *Nat. Commun.* **10**, 631 (2019).

35. Liu T, Wang S, Zhang Q, Chen L, Hu W, Li CM. Ultrasmall Ru2P nanoparticles on graphene: A highly efficient hydrogen evolution reaction electrocatalyst in both acidic and alkaline media. *Chem. Commun.* **54**, 3343-3346 (2018).

36. Xu C*, et al.* Facile synthesis of effective ru nanoparticles on carbon by adsorption-low temperature pyrolysis strategy for hydrogen evolution. *J. Mater. Chem. A* **6**, 14380-14386 (2018).

37. Cheng X*, et al.* Well-defined Ru nanoclusters anchored on carbon: Facile synthesis and high electrochemical activity toward alkaline water splitting. *ACS Sustainable Chem. Eng.* **6**, 11487-11492 (2018).

38. Wang H*, et al.* Nitrogen-doped carbon-stabilized Ru nanoclusters as excellent catalysts for hydrogen production. *ACS Sustainable Chem. Eng.* **7**, 1178-1184 (2019).

39. Yu J*, et al.* Bigger is surprisingly better: Agglomerates of larger RuP nanoparticles outperform benchmark Pt nanocatalysts for the hydrogen evolution reaction. *Adv. Mater.* **30**, 1800047 (2018).

40. Zhang J*, et al.* Ruthenium/nitrogen-doped carbon as an electrocatalyst for efficient hydrogen evolution in alkaline solution. *J. Mater. Chem. A* **5**, 25314-25318 (2017).

41. Li F, Han G-F, Noh H-J, Ahmad I, Jeon I-Y, Baek J-B. Mechanochemically assisted synthesis of a Ru catalyst for hydrogen evolution with performance superior to Pt in both acidic and alkaline media. *Adv. Mater.* **30**, 1803676 (2018).

42. Lu Q*, et al.* Synthesis of hierarchical 4h/fcc Ru nanotubes for highly efficient hydrogen evolution in alkaline media. *Small* **14**, 1801090 (2018).

43. Wang Q, Ming M, Niu S, Zhang Y, Fan G, Hu J-S. Scalable solid-state synthesis of highly dispersed uncapped metal (Rh, Ru, Ir) nanoparticles for efficient hydrogen evolution. *Adv. Energy Mater.* **8**, 1801698 (2018).

44. Yu J, Guo Y, Miao S, Ni M, Zhou W, Shao Z. Spherical ruthenium disulfide-sulfur-doped graphene composite as an efficient hydrogen evolution electrocatalyst. *ACS Applied Materials & Interfaces* **10**, 34098-34107 (2018).

45. Yang K*, et al.* Ultrasmall Ru/Cu-doped RuO_2_ complex embedded in amorphous carbon skeleton as highly active bifunctional electrocatalysts for overall water splitting. *Small* **14**, 1803009 (2018).

46. Liu Y*, et al.* Ru modulation effects in the synthesis of unique rod-like Ni@Ni2P–Ru heterostructures and their remarkable electrocatalytic hydrogen evolution performance. *J. Am. Chem. Soc.* **140**, 2731-2734 (2018).

47. Wang J, Wei Z, Mao S, Li H, Wang Y. Highly uniform Ru nanoparticles over N-doped carbon: Ph and temperature-universal hydrogen release from water reduction. *Energy Environ. Sci.* **11**, 800-806 (2018).

48. Song Q, Qiao X, Liu L, Xue Z, Huang C, Wang T. Ruthenium@N-doped graphite carbon derived from carbon foam for efficient hydrogen evolution reaction. *Chem. Commun.* **55**, 965-968 (2019).

49. Gou W, Li J, Gao W, Xia Z, Zhang S, Ma Y. Downshifted d-band center of Ru/MWCNTs by turbostratic carbon nitride for efficient and robust hydrogen evolution in alkali. *ChemCatChem* **11**, 1970-1976 (2019).

50. Yuan C-Z*, et al.* Molecule-assisted synthesis of highly dispersed ultrasmall RuO_2_ nanoparticles on nitrogen-doped carbon matrix as ultraefficient bifunctional electrocatalysts for overall water splitting. *ACS Sustainable Chem. Eng.* **6**, 11529-11535 (2018).

51. Gao K*, et al.* Ru nanodendrites composed of ultrathin fcc/hcp nanoblades for the hydrogen evolution reaction in alkaline solutions. *Chem. Commun.* **54**, 4613-4616 (2018).

52. Xu Y*, et al.* Ultrathin nitrogen-doped graphitized carbon shell encapsulating CoRu bimetallic nanoparticles for enhanced electrocatalytic hydrogen evolution. *Nanotechnology* **29**, 225403 (2018).

53. Zhu Y*, et al.* Unusual synergistic effect in layered Ruddlesden−Popper oxide enables ultrafast hydrogen evolution. *Nat. Commun.* **10**, 149 (2019).

54. Chi J-Q*, et al.* Hydrogen evolution activity of ruthenium phosphides encapsulated in nitrogen- and phosphorous-codoped hollow carbon nanospheres. *ChemSusChem* **11**, 743-752 (2018).

55. Bhowmik T, Kundu MK, Barman S. Growth of one-dimensional RuO_2_ nanowires on g-carbon nitride: An active and stable bifunctional electrocatalyst for hydrogen and oxygen evolution reactions at all pH values. *ACS Applied Materials & Interfaces* **8**, 28678-28688 (2016).

56. Lu Q*, et al.* Crystal phase-based epitaxial growth of hybrid noble metal nanostructures on 4h/fcc Au nanowires. *Nat. Chem.* **10**, 456-461 (2018).

57. Wang J, Han L, Huang B, Shao Q, Xin HL, Huang X. Amorphization activated ruthenium-tellurium nanorods for efficient water splitting. *Nat. Commun.* **10**, 5692 (2019).

58. Fan J*, et al.* Interstitial hydrogen atom modulation to boost hydrogen evolution in Pd-based alloy nanoparticles. *ACS Nano* **13**, 12987-12995 (2019).

59. Li H*, et al.* Amorphous nickel-cobalt complexes hybridized with 1T-phase molybdenum disulfide via hydrazine-induced phase transformation for water splitting. *Nat. Commun.* **8**, 15377 (2017).

60. Li H*, et al.* Activating and optimizing MoS_2_ basal planes for hydrogen evolution through the formation of strained sulphur vacancies. *Nat. Mater.* **15**, 48-53 (2016).

61. Geng X*, et al.* Pure and stable metallic phase molybdenum disulfide nanosheets for hydrogen evolution reaction. *Nat. Commun.* **7**, 10672 (2016).

62. Zang Y*, et al.* Tuning orbital orientation endows molybdenum disulfide with exceptional alkaline hydrogen evolution capability. *Nat. Commun.* **10**, 1217 (2019).

63. Zhao D*, et al.* Synergistically interactive pyridinic-N–MoP sites: Identified active centers for enhanced hydrogen evolution in alkaline solution. *Angew. Chem. Int. Ed.* **59**, 8982-8990 (2020).

64. Lu XF, Yu L, Lou XW. Highly crystalline Ni-doped FeP/carbon hollow nanorods as all-pH efficient and durable hydrogen evolving electrocatalysts. *Sci. Adv.* **5**, eaav6009 (2019).

65. Yan H*, et al.* Holey reduced graphene oxide coupled with an Mo_2_N–Mo_2_C heterojunction for efficient hydrogen evolution. *Adv. Mater.* **30**, 1704156 (2018).

66. Liang H-W, Brüller S, Dong R, Zhang J, Feng X, Müllen K. Molecular metal–N_x_ centres in porous carbon for electrocatalytic hydrogen evolution. *Nat. Commun.* **6**, 7992 (2015).

67. Wu HB, Xia BY, Yu L, Yu X-Y, Lou XW. Porous molybdenum carbide nano-octahedrons synthesized via confined carburization in metal-organic frameworks for efficient hydrogen production. *Nat. Commun.* **6**, 6512 (2015).
